# The generalized ratios intrinsic dimension estimator

**DOI:** 10.1038/s41598-022-20991-1

**Published:** 2022-11-21

**Authors:** Francesco Denti, Diego Doimo, Alessandro Laio, Antonietta Mira

**Affiliations:** 1 Department of Statistics, Università Cattolica del Sacro Cuore, Milan, Italy; 2 SISSA, Via Bonomea 265, Trieste, Italy; 3 ICTP, Strada Costiera 11, 34151, Trieste, Italy; 4 Faculty of Economics, Università della Svizzera italiana, Lugano, Switzerland; 5 University of Insubria, Varese, Italy

**Keywords:** Computer science, Statistics

## Abstract

Modern datasets are characterized by numerous features related by complex dependency structures. To deal with these data, dimensionality reduction techniques are essential. Many of these techniques rely on the concept of intrinsic dimension (id), a measure of the complexity of the dataset. However, the estimation of this quantity is not trivial: often, the id depends rather dramatically on the scale of the distances among data points. At short distances, the id can be grossly overestimated due to the presence of noise, becoming smaller and approximately scale-independent only at large distances. An immediate approach to examining the scale dependence consists in decimating the dataset, which unavoidably induces non-negligible statistical errors at large scale. This article introduces a novel statistical method, Gride, that allows estimating the id as an explicit function of the scale without performing any decimation. Our approach is based on rigorous distributional results that enable the quantification of uncertainty of the estimates. Moreover, our method is simple and computationally efficient since it relies only on the distances among data points. Through simulation studies, we show that Gride is asymptotically unbiased, provides comparable estimates to other state-of-the-art methods, and is more robust to short-scale noise than other likelihood-based approaches.

## Introduction

In recent years, we have witnessed an unimaginable growth in data production. From personalized medicine to finance, datasets characterized by a large number of features are ubiquitous in modern data analyses. The availability of these high-dimensional datasets poses novel and engaging challenges for the statistical community, called to devise new techniques to extract meaningful information from the data in a limited amount of computational time and memory. Fortunately, data contained in high-dimensional embeddings can often be described by a handful of variables: a subset of the original ones or a combination—not necessarily linear—thereof. In other words, one can effectively map the features of a dataset onto spaces of much lower dimension, typically nonlinear manifolds^1^. Estimating the dimensionality of these manifolds is of paramount importance. We will call this quantity the *intrinsic dimension* (id from now on) of a dataset, i.e., the number of relevant coordinates needed to describe the data-generating process accurately^2^.

Many definitions of id have been proposed in the literature since this concept has been investigated in a wide range of disciplines ranging from mathematics, physics, and engineering to computer science and statistics. For example, Fukanaga^3^ expressed the id as the minimum number of parameters needed to describe the essential characteristics of a system accurately. For^4^, the id is the dimension of the subspace in which the data are entirely located, without information loss. An alternative definition, that exploits the language of pattern recognition, is provided by^5^. In this framework, a set of points is viewed as a uniform sample obtained from a distribution over an unknown smooth (or locally smooth) manifold structure (its support),eventually embedded in a higher-dimensional space through a nonlinear smooth mapping. Thus, the id represents the topological dimension of the manifold. All these definitions are useful for delineating different aspects of the multi-faceted concept that is the id.

The literature on statistical methods for dimensionality reduction and id estimation is extraordinarily vast and heterogeneous. We refer to^5,6^ for comprehensive reviews of state-of-the-art methods, where the strengths and weaknesses of numerous methodologies are outlined and compared. Generally, methods for the estimation of the $\texttt {id }$ can be divided into two main families: *projective methods* and *geometric methods*.

On the one hand, *projective methods* estimate the low-dimensional embedding of interest through transformations of the data, which can be linear, such as Principal Component Analysis (PCA)^7^ and its Probabilistic^8^, Bayesian^9^, and Sparse^10^ extensions; or nonlinear, as Local Linear Embedding^11^, Isomap^12^, and others^13,14^. See also^15^ and the references therein.

On the other hand, *geometric methods* rely on the topology of a dataset, exploiting the properties of distances among data points. Within this family, we can distinguish among *fractal methods*^16^, *graphical methods*^17,18^, and *methods based on nearest neighbor distances* (e.g., IDEA^19^) *and angles* (e.g., DANCo^20^). We will focus on the latter category, which is directly related to our proposal.

Nearest neighbors (NNs) methods rely on the assumption that points close to each other can be considered as uniformly drawn from *d*-dimensional balls (hyperspheres). More formally, consider a generic data point $\varvec{x}$ and denote with ${\mathcal {B}}_{d}(\varvec{x}, r)$ a hypersphere, characterized by a small radius $r \in {\mathbb {R}}^{+}$, centered in $\varvec{x}$. Let $\rho (\varvec{x})$ be the density function of the points in ${\mathbb {R}}^{d}$. Intuitively, the proportion of points of a given sample of size *n* from $\rho (\varvec{x})$ that falls into ${\mathcal {B}}(\varvec{x}, r)$ is approximately $\rho (\varvec{x})$ times the volume of the ball. This intuition gives rise to the following formal relationship: $\frac{k}{n} \approx \rho (\varvec{x})\, \omega _{d}\, r^{d}$. Here, *k* is the number of NNs of $\varvec{x}$ within the hypersphere ${\mathcal {B}}_d(\varvec{x}, r)$, while $\omega _{d}$ is the volume of the *d*-dimensional unit hypersphere in ${\mathbb {R}}^{d}$. If, in the previous relationship, the density is assumed to be constant, one can estimate the id as a function of the average of the distances among the sample points and their respective *k*-th NN^21^. This type of approach gives rise to the question on how to effectively select *k*, the number of considered NNs.

From a different perspective, various authors adopted model-based frameworks for manifold learning and id estimation. One possible approach is to specify a model for the distribution of the distances among the data points. Amsaleg et al.^22^, exploiting results from^23^, suggested modeling the distances as a Generalized Pareto distribution since they showed that a (local) id can be recovered, asymptotically, as a function of its parameters. In a Bayesian framework, Duan and Dunson^24^ proposed modeling the pairwise distances among data points to coherently estimate a clustering structure. Furthermore, some model-based methods to explore the topology of datasets have recently been developed, pioneered by the likelihood approach discussed in^1^. Mukhopadhyay et al.^25^ used Fisher-Gaussian kernels to estimate densities of data embedded in nonlinear subspaces. Li et al.^26^ proposed to learn the structure of latent manifolds by approximating them with spherelets instead of locally linear approximation, developing a spherical version of PCA. In the same spirit, Li and Dunson^27^ applied this idea to the classification of data lying on complex, nonlinear, overlapping, and intersecting supports. Similarly, Li and Dunson^28^ proposed to use the spherical PCA to estimate a geodesic distance matrix, which takes into account the structure of the latent embedding manifolds, and created a spherical version of the *k*-medoids algorithm^29^.

Alternatively, Gomtsyan et al.^30^ directly extended the maximum likelihood estimator (MLE) by^1^ proposing a geometry-aware estimator to correct the negative bias that often plagues MLE approaches in high dimensions. The geometric properties of a dataset are also exploited by the ESS estimator^31^, which is based on the evaluation of simplex volumes spanned by data points. Finally, Serra and Mandjes^32^ and Qiu et al.^33^ estimated the id via random graph models applied to the adjacency matrices among data points, recovered by connecting observations whose distances do not exceed a certain threshold.

This paper introduces a likelihood-based approach to derive a novel $\texttt {id }$ estimator. Our result stems from the geometrical probabilistic properties of the NNs distances. Specifically, we build on the two nearest neighbors (TWO-NN) estimator, recently introduced by^2^. Similarly to^1,34^, the TWO-NN is a model-based id estimator derived from the properties of a Poisson point process, whose realizations occur on a manifold of dimension *d*. Facco et al.^2^ proved that the ratio of distances between the second and first NNs of a given point is Pareto distributed with unitary scale parameter and shape parameter precisely equal to *d*. To estimate the id, they suggested fitting a Pareto distribution to a proper transformation of the data. Their result holds under mild assumptions on the data-generating process, which we will discuss in detail.

We extend the TWO-NN theoretical framework by deriving closed-form distributions for the product of consecutive ratios of distances and, more importantly, for the ratio of distances among NNs of generic order.

These theoretical derivations have relevant practical consequences. By leveraging our distributional results, we attain an estimator that is more robust to the noise present in a dataset, as we will show with various simulation studies. Moreover, the new estimator allows the investigation of the $\texttt {id }$ evolution as a function of the distances among NNs. Monitoring this evolution is beneficial for two reasons. First, it is a way to examine how the $\texttt {id }$ depends on the size of the neighborhood at hand. Second, as the size of the neighborhood increases, our estimator can reduce the bias induced by potential measurement noise. Finally, the principled derivation of our results enables the immediate specification of methods to perform uncertainty estimation.

The article is organized as follows. Section “Likelihood-based TWO-NN estimators” briefly introduces the TWO-NN modeling framework developed by^2^ and discusses the MLE and Bayesian alternatives. In “Gride, the generalized ratios intrinsic dimension estimator”, we contribute to the Poisson point process theory by providing closed-form distributions for functions of distances between a point and its NNs. We exploit these results to devise a new estimator for the $\texttt {id }$ of a dataset that we name Gride. Section “Results” presents several numerical experiments that illustrate the behavior of Gride. We compare our proposal with other relevant estimators in terms of estimated values, robustness to noise, and computational cost. In “Discussion”, we discuss possible future research directions. The interested reader is also referred to the Supplementary Material, where we report the proofs of our theoretical results, along with extended simulation studies.

## Methods

### Likelihood-based TWO-NN estimators

In this section, we briefly introduce the modeling framework that led to the development of the TWO-NN estimator, propose a maximum likelihood and Bayesian counterparts, and discuss its shortcomings when applied to noisy datasets. More details about this estimator and its assumptions are deferred to the Supplementary Material.

Consider a dataset $\varvec{X}=\{\varvec{x}_i\}_{i=1}^n$ composed of *n* observations measured over *D* distinct features, i.e., $\varvec{x}_i\in {\mathbb {R}}^D$, for $i=1,\ldots ,n$. Denote with $\Delta :{\mathbb {R}}^D\times {\mathbb {R}}^D \rightarrow {\mathbb {R}}^+$ a generic distance function between pairs of elements in ${\mathbb {R}}^D$. We assume that the dataset $\varvec{X}$ is a particular realization of a Poisson point process characterized by density function (that is, normalized intensity function) $\rho \left( \varvec{x}\right)$. We also suppose that the density of the considered stochastic process has its support on a manifold of unknown intrinsic dimension $d\le D$. We expect, generally, that $d<<D$.

For any fixed point $\varvec{x}_i$, we sort the remaining $n-1$ observations according to their distance from $\varvec{x}_i$ by increasing order. Let us denote with $\varvec{x}_{(i,l)}$ the *l*-th NN of $\varvec{x}_i$ and with $r_{i,l}=\Delta (\varvec{x}_{i},\varvec{x}_{(i,l)})$ their distance, with $l=1,\ldots , n-1$. For notation purposes, we define $\varvec{x}_{i,0}\equiv \varvec{x}_{i}$ and $r_{i,0}=0$.

A crucial quantity in this context is the *volume of the hyperspherical shell enclosed between two successive neighbors of* $\varvec{x}_i$, defined as

1 $$\begin{aligned} v_{i,l}=\omega _{d}\left( r_{i,l}^{d}-r_{i,l-1}^{d}\right) , \quad \quad \text {for }l =1,\ldots ,n-1,\text { and }i=1,\dots ,n, \end{aligned}$$

where *d* is the dimension of the space in which the points are embedded (the id) and $\omega _{d}$ is the volume of the *d*-dimensional sphere with unitary radius. We also assume that the density function $\rho$ is constant. Under these premises, we have $v_{i,l}\sim Exp(\rho )$, for $l =1,\ldots ,n-1,$ and $i=1,\dots ,n$.

#### Theorem 2.1

^2^ Consider a distance function $\Delta$ taking values in ${\mathbb {R}}^+$ defined among the data points $\{\varvec{x}_i\}_{i=1}^n$, which are a realization of a Poisson point process with constant density $\rho$. Let $r_{i,l}$ be the value of this distance between observation *i* and its *l*-th NN. Then,

2 $$\begin{aligned} \mu _i = \dfrac{r_{i,2}}{r_{i,1}} \sim Pareto(1,d), \quad \quad \mu _i \in \left( 1,+\infty \right) . \end{aligned}$$

An alternative proof for this result is reported in the Supplementary Material.

We remark that, while the theorem can be proven only if the density $\rho$ is constant, the result and the $\texttt {id }$ estimator are empirically valid as long as the density is approximately constant on the scale defined by the distance of the second NN $r_{i,2}$. We refer to this weakened assumption as *local homogeneity*.

The TWO-NN estimator treats the ratios in $\varvec{\mu }=\{\mu _i\}_{i=1}^n$ as *independent*, $i=1,\ldots ,n$, and estimates the global $\texttt {id }$ employing a least-squares approach. In detail, Facco et al.^2^ proposed to consider the cumulative distribution function (c.d.f.) of each ratio $\mu _i$ given by $F({\mu _i})= (1-\mu _i^{-d})$, and to linearize it into $\log (1-F({\mu _i}))=-d\log (\mu _i)$. Then, a linear regression with no intercept is fitted to the pairs $\{-\log (1-{\tilde{F}}(\mu _{(i)})),\log (\mu _{(i)}) \}_{i=1}^n$, where ${\tilde{F}}(\mu _{(i)})$ denotes the empirical c.d.f. of the sample $\varvec{\mu }$ sorted by increasing order. To improve the estimation, the authors also suggested discarding the ratios $\mu _i$’s that fall above a given high percentile (e.g., 90%), usually generated by observations that fail to comply with the local homogeneity assumption. Since it is based on a simple linear regression, the TWO-NN estimator provides a fast and accurate estimation of the $\texttt {id }$, even when the sample size is large. Nonetheless, from (2) we can immediately derive the corresponding maximum likelihood estimator (MLE) and the posterior distribution of *d* within a Bayesian setting. First, let us discuss the MLE and the relative confidence intervals (CI). For the shape parameter of a Pareto distribution, the (unbiased) MLE is given by:

3 $$\begin{aligned} {\hat{d}} = \frac{n-1}{\sum _{i}^n\log (\mu _i)}. \end{aligned}$$

Moreover, ${\hat{d}}/d \sim IG(n,(n-1))$, where *IG* denotes an Inverse-Gamma distribution. Therefore, the corresponding confidence interval (CI) of level $1-\alpha$ is given by

4 $$\begin{aligned} CI(d,1-\alpha )=\left[ \frac{{\hat{d}}}{q^{1-\alpha /2}_{IG_{n,(n-1)}}}; \frac{{\hat{d}}}{q^{\alpha /2}_{IG_{n,(n-1)}}}\right] , \end{aligned}$$

where $q^{\alpha /2}_{IG}$ denotes the quantile of order $\alpha /2$ of an Inverse-Gamma distribution.

Alternatively, to carry out inference under the Bayesian approach we specify a prior distribution on the parameter *d*. The most convenient prior choice is $d\sim Gamma(a,b)$ because of its conjugacy property. In this case, it is immediate to derive the posterior distribution of the id:

5 $$\begin{aligned} d|\varvec{\mu } \sim Gamma\left( a+n, b+\sum _{i=1}^n \log (\mu _i)\right) . \end{aligned}$$

Under the Bayesian paradigm, we obtain the credible intervals by taking the relevant quantiles of the posterior distribution. Moreover, one can immediately derive the posterior predictive distribution

6 $$\begin{aligned} p({\tilde{\mu }}|\varvec{\mu })= \frac{a^*}{b^*\;{\tilde{\mu }}}\left( 1+\frac{\log ({\tilde{\mu }})}{b^*}\right) ^{-a^*-1}, \text { with } \, {\tilde{\mu }} \in \left( 1,+\infty \right) , \end{aligned}$$

where $a^*=a+n$ and $b^*=b+\sum _{i=1}^n \log (\mu _i)$. The posterior predictive distribution is useful to assess the model’s goodness of fit. For example, one can compute the discrepancy between synthetic data generated from the distribution in (6) and the dataset at hand to assess the validity of the assumed data-generating mechanism^35^. From Eq. (6), it can be easily shown that the posterior predictive law for $\log ({\tilde{\mu }})$ follows a $Lomax(a^{*},b^{*})$ distribution, for which samplers are readily available.

The derivations in (3)–(5) lead to alternative ways to estimate—by point or confidence/credible intervals—the $\texttt {id }$ within the TWO-NN model enabling immediate uncertainty quantification, an aspect that was not developed in detail in^2^.

**Figure 1:**
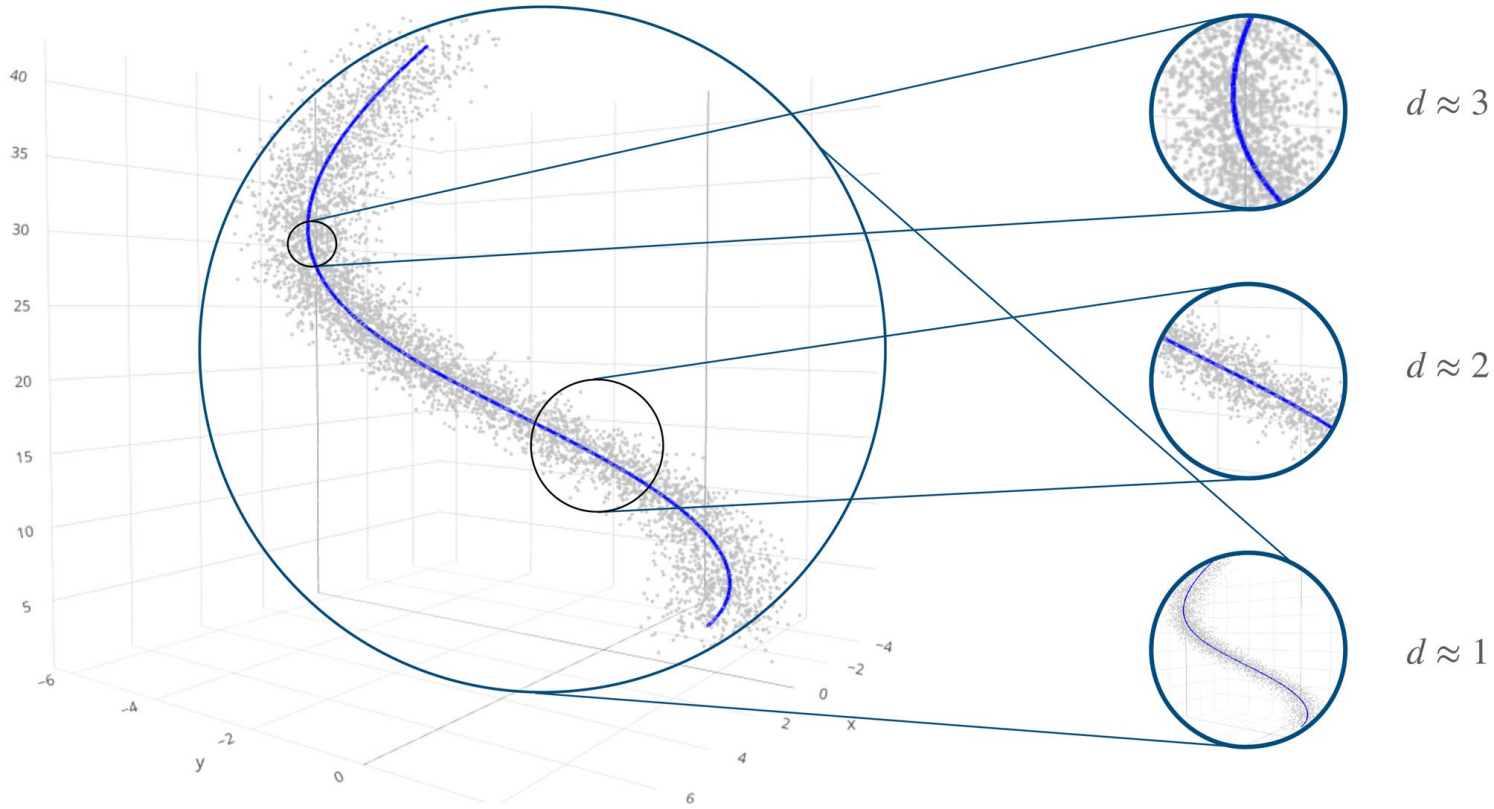
Three-dimensional Spiral dataset, with $n=5000$, ${\bar{S}}=1$, $\sigma ^2_x=\sigma ^2_y=0.5$, and $\sigma ^2_z=1$. The resulting data points are displayed on the left. On the right, we show how observing the data at different scales can produce different insights regarding the dimensionality of the dataset.

**Table 1:** MLE point estimates and confidence intervals computed on the Spiral dataset ($d=1$) with the TWO-NN and Gride estimators.

$d=1$	TWO-NN				$\texttt {Gride}(n_1,n_2)$			
	$c=1$	$c=0.20$	$c=0.01$	$c=0.001$	(2, 4)	(100, 200)	(250, 500)	(750, 1500)
Lower Bound	2.910	2.811	2.682	0.692	2.953	2.688	1.557	0.996
Estimate	2.991	2.990	3.541	1.706	3.014	2.699	1.560	0.997
Upper Bound	3.075	3.182	4.681	4.368	3.074	2.710	1.562	0.998
Bias	1.991	1.990	2.541	0.706	2.014	1.699	0.560	-0.003
CI width	0.165	0.371	1.999	3.676	0.121	0.022	0.005	0.002

Different levels of decimation *c* and different NN orders $(n_1,n_2)$ are presented across the columns. The last two rows report the bias in the estimates, ${\hat{d}}-d$, and the width of the CIs.

The TWO-NN modeling framework presents a potential shortcoming: it does not account for the presence of noise in the data. Measurement errors can significantly impact the estimates since the $\texttt {id }$ estimators are sensitive to different sizes of the considered neighborhood. As an example, consider a dataset of *n* observations measured in ${\mathbb {R}}^3$ created as follows. The first two coordinates are obtained from the spiral defined by the parametric equations $x=u \cos (u+2\pi )$ and $y= u\sin (u+2\pi )$, where $u = 2\pi \sqrt{u_0}$ and $u_0$ is attained from an evenly-spaced grid of *n* points over the support $\left[ \frac{1}{4\pi },{\bar{S}}\right]$. The third coordinate is defined as a function of the previous two, $z = x^2 + y^2$. Gaussian random noise (with standard deviations $\sigma _x,\,\sigma _y$, and $\sigma _z$) is added to all three coordinates. We simulated a first Spiral dataset setting $n=5000$, ${\bar{S}}=1$, $\sigma _x=\sigma _y=0.5$, and $\sigma _z=1$. A three-dimensional depiction of the resulting dataset is reported in the left part of Fig. 1. The value of the $\texttt {id }$ estimated with the TWO-NN model is 2.99. However, $u_0$ is the only free variable since the three coordinates are deterministic functions of $u_0$. Therefore, only one degree of freedom is used in the data generating process. In other words, the true $\texttt {id }$ is 1, and the noise at short scale misleads the TWO-NN estimator. For a visual example of how the $\texttt {id }$ may change with the size of the considered neighborhood, see the right part of Fig. 1. As a strategy to mitigate the local noise effect, Facco et al.^2^ proposed to subsample the dataset at hand and consider only a fraction *c* of the points. By doing this, we effectively extend the average size of the neighborhood considered by the estimator. Although this decimation strategy helps understand how the TWO-NN is affected by the resolution of the considered neighborhood, it comes at a critical cost in terms of statistical power. As the value of *c* decreases, this procedure discards the majority of the data points. Moreover, little heuristic on how to fix an optimal value is available. Facco et al.^2^ proposed to monitor the $\texttt {id }$ evolution as a function of *c*, looking for a plateau in the estimates. Thus, the best value for *c* would be the highest proportion such that the $\texttt {id }$ is close to the plateau values.

In the next section, we will introduce the Gride, which is based on ratios of NNs distances of order higher than the second. Let us denote the orders of the considered NNs with $n_1$ and $n_2$, respectively. This novel estimator can go beyond the local reach of the TWO-NN, effectively reducing the impact of noise on the id estimate. Moreover, by increasing the order of the considered NNs, we can monitor how the id estimate changes as a function of the neighborhood size without discarding any data point. As a preliminary result, we compare the performance of Gride with the decimated TWO-NN on the Spiral dataset. We report in Table 1 the point estimates (obtained via MLE) and confidence intervals, along with the corresponding bias and interval width. The first four columns show the results for the TWO-NN estimator applied to a fraction $c\in \{1;\,0.20;\,0.01;\,0.001\}$ of the original dataset. The remaining four columns contain the results for Gride with different NN orders: $(n_1,n_2)\in \{(2,4);\,(100,200);\,(250,500);\,(750,1500)\}$. We aim to monitor the evolution of the estimate as a function of the NN orders to assess the model’s sensitivity to the noise.

On the one hand, the TWO-NN estimator applied to a decimated dataset leads to reasonable point estimates when minimal values of *c* are considered. However, this comes at the price of greater uncertainty, which is reflected by the wider confidence intervals. Gride, on the other hand, escapes the positive bias induced by the noise for large values of $n_1$ and $n_2$ while maintaining narrow confidence intervals. Note that low values of *c* and high values of $n_2$ induce the TWO-NN and Gride, respectively, to cover broader neighborhoods. However, the smaller uncertainty of Gride highlights that our method does not have to discard any information to reach this goal. This preliminary result suggests that, by extending the orders of NNs distances that we consider, Gride can escape the short, “local reach” of the TWO-NN model, which is extremely sensitive to data noise. Thus, extending the neighborhood of a point to further NNs allows extracting meaningful information about the topology and the dataset’s structure at different distance resolutions.

### Gride, the generalized ratios intrinsic dimension estimator

In this section, we develop novel theoretical results that contribute to the Poisson point processes theory. We will then exploit these results to devise a better estimator for *d*. In detail, we first extend the distributional results of “Likelihood-based TWO-NN estimators” providing closed-form distributions for vectors of consecutive ratios of distances. Then, building upon that, we move a step further and derive the closed-form expression for the distribution of ratios of NNs of generic order.

#### Distribution of consecutive ratios, generic ratios, and related estimators

Consider the same setting introduced in the previous section and define $V_{i,l} = \omega _d \, r^d_{i,l}$ as the volume of the hypersphere centered in $\varvec{x}_i$ with radius equal to the distance between $\varvec{x}_i$ and its *l*-th NN. Because of their definitions, for $l=2,\ldots ,L$, we have that $v_{i,l}$ and $V_{i,l-1}=v_{i,1}+\cdots +v_{i,l-1}$ are independent. Moreover, $V_{i,l}\sim Erlang(1,l-1)$. Then, we can write

7 $$\begin{aligned} \frac{v_{i,l}}{V_{i,l-1}} = \frac{\omega _d \left( r_{i,l}^d-r_{i,l-1}^d \right) }{\omega _d r^d_{i,l-1}} =\left( \frac{r_{i,l} }{ r_{i,l-1}}\right) ^d-1, \end{aligned}$$

which can be re-expressed as

8 $$\begin{aligned} \mu _{i,l}=\frac{r_{i,l} }{ r_{i,l-1}} = \left( \frac{v_{i,l}}{V_{i,l-1}}+1\right) ^{1/d}. \end{aligned}$$

Given these premises, the following theorem holds.

##### Theorem 2.2

Consider a distance $\Delta$ taking values in ${\mathbb {R}}^+$ defined among the data points $\{\varvec{x}_i\}_{i=1}^n$, which are realizations of a Poisson point process with constant density $\rho$. Let $r_{i,l}$ be the value of the distance between observation *i* and its *l*-th NN. Define $\mu _{i,l} =r_{i,l} / r_{i,l-1}$. It follows that

9 $$\begin{aligned} \begin{aligned} \mu _{i,l}&\sim Pareto\,(1,(l-1)d), \quad \text { for }\quad l = 2,\ldots ,L. \end{aligned} \end{aligned}$$

Moreover, the elements of the vector $\varvec{\mu }_{i,L}=\{ \mu _{i,l} \}_{l=2}^L$ are jointly independent.

The proof is deferred to the Supplementary Material. Theorem 2.2 provides a way to characterize the distributions of consecutive ratios of distances. Remarkably, given the homogeneity assumption, the different ratios are all independent. Building on the previous statements, we can derive more general results about the distances among NNs from a Poisson point process realization. The following theorem characterizes the distribution of the ratio of distances from two NNs of generic order. It will be the foundation of the estimator that we propose in this paper.

##### Theorem 2.3

Consider a distance $\Delta$ taking values in ${\mathbb {R}}^+$ defined among the data points $\{\varvec{x}_i\}_{i=1}^n$, which are realizations of a Poisson point process with constant density $\rho$. Let $r_{i,l}$ be the value of this distance between observation *i* and its *l*-th NN. Consider two integers $1\le n_1<n_2$ and define ${\dot{\mu }}=\mu _{i,n_1,n_2} = r_{i,n_2} / r_{i,n_1}$. The random variable ${\dot{\mu }}$ is characterized by density function

10 $$\begin{aligned} f_{\mu _{i,n_1,n_2}}({\dot{\mu }})= \frac{d({\dot{\mu }}^d-1)^{n_2-n_1-1}}{{\dot{\mu }}^{(n_2-1)d+1}B(n_2-n_1,n_1)}, \quad {\dot{\mu }}>1, \end{aligned}$$

where $B(\cdot ,\cdot )$ denotes the Beta function. Moreover, ${\dot{\mu }}$ has *k*-th moment given by

11 $$\begin{aligned} {\mathbb {E}}\left[ {\dot{\mu }}^k\right] =\frac{B(n_2-n_1,n_1-k/d)}{B(n_2-n_1,n_1)}. \end{aligned}$$

The proof is given in the Supplementary Material. Moreover, we also report a figure with some examples of the shapes of the density functions defined in Eq. (10). We now state some important remarks.

##### *Remark 1*

Given the expression of the generic moment of ${\dot{\mu }}$, we can derive its expected value and variance:

12 $$\begin{aligned} {\mathbb {E}}\left[ {\dot{\mu }}\right] = \frac{B(n_2-n_1,n_1-1/d)}{B(n_2-n_1,n_1)}\quad \text {and} \quad {\mathbb {V}}\left[ {\dot{\mu }}\right] =\frac{B(n_2-n_1,n_1-2/d)}{B(n_2-n_1,n_1)} -\frac{B(n_2-n_1,n_1-1/d)^2}{B(n_2-n_1,n_1)^2}, \end{aligned}$$

both well-defined when $d>2$. From the first equation, it is straightforward to derive an estimator based on the method of moments.

##### *Remark 2*

Formula (10) can be specialized to the case where $n_1=n_0$ and $n_2=2n_0$. We obtain

13 $$\begin{aligned} f_{\mu _{i,n_0,2n_0}}({\dot{\mu }})= \frac{(2n_0-1)!}{(n_0-1)!^2} \cdot \frac{d({\dot{\mu }}^d-1)^{n_0-1}}{{\dot{\mu }}^{(2n_0-1)d+1}} = \frac{d({\dot{\mu }}^d-1)^{n_0-1}}{B(n_0,n_0)\cdot {\dot{\mu }}^{(2n_0-1)d+1}} , \quad {\dot{\mu }}>1. \end{aligned}$$

##### *Remark 3*

The result in Eq. (9) can be derived as a special case of formula (10). Consequently, we can say the same for the TWO-NN model in Eq. (2). Specifically, if we set $n_1=n_0$ and $n_2=n_0+1$, we obtain

14 $$\begin{aligned} f_{\mu _{i,n_0,n_0+1}}({\dot{\mu }})= n_0 d {\dot{\mu }}^{-n_0d-1}, \quad {\dot{\mu }}>1, \end{aligned}$$

which is the density of a $Pareto(1,n_0d)$ distribution.

##### *Remark 4*

Given the previous results, it is also possible to show that, within our theoretical framework, the joint density of the random distances between a point and its first *L* NNs follows a Generalized Gamma distribution. We report a formal statement of this result and its proof in the Supplementary Material.

The distributions reported in Eqs. (10) and (13) allow us to devise a novel estimator for the $\texttt {id }$ parameter based on the properties of the distances measured between a point and two of its NNs of generic order. We name this method the *Generalized ratios* $\texttt {id }$ *estimator* (Gride). From Eq. (10), by assuming that the *n* observations are independent, we derive the expression of the log-likelihood:

15 $$\begin{aligned} \log {\mathcal {L}}=n\log (d)+(n_2-n_1-1)\sum _i\log ({\dot{\mu }}^d_i-1) - \log (B(n_2-n_1,n_1)) - ((n_2-1)d+1)\sum _i\log ({\dot{\mu }}_i). \end{aligned}$$

Following a maximum likelihood approach, we estimate *d* by finding the root of the following score function:

$$\begin{aligned} \frac{\partial \log {\mathcal {L}}}{\partial d}=\frac{n}{d}+(n_2-n_1-1)\sum _i \frac{{\dot{\mu }}^d_i\log ({\dot{\mu }}_i)}{{\dot{\mu }}^d_i-1}-(n_2-1)\sum _i \log ({\dot{\mu }}_i)=0. \end{aligned}$$

This equation cannot be solved in closed-form, but the second derivative of the log-likelihood function $\log {\mathcal {L}}$ for *n* observations is always negative on the entire parameter space $d\in \left[ 1,+\infty \right)$:

$$\begin{aligned} \frac{\partial ^2 \log {\mathcal {L}}}{\partial d^2} = -\frac{n}{d^2}-(n_2-n_1-1)\sum _i \frac{{\dot{\mu }}^d_i\log ({\dot{\mu }}_i)^2}{({\dot{\mu }}^d_i-1)^2} <0. \end{aligned}$$

Therefore, the log-likelihood function is concave, and univariate numerical optimization routines can obtain the MLE. Moreover, one can exploit numerical methods for uncertainty quantification: for example, one can estimate the confidence intervals with parametric bootstrap^36^.

A more straightforward alternative estimator can be devised by setting $n_2=n_1+1$ and leveraging on the consecutive ratios independence result presented in Theorem 2.3. In this specific case, we can derive an estimator that is the direct extension of the MLE version of the TWO-NN:

16 $$\begin{aligned} {\hat{d}}_L = \frac{n(L-1)-1}{\sum _{i=1}^n\sum _{l=2}^L (l-1)\log (\mu _{i,l})}, \end{aligned}$$

by focusing on the properties of consecutive ratios of distances contained in the vectors $\varvec{\mu }_{i,L}$, for $i=1,\ldots , n$.

The estimator in (16) has variance ${\mathbb {V}}\left[ {\hat{d}}_L\right] =d^2/(n(L-1)-2)$ which is smaller than the variance of the MLE estimator in (3), that is recovered when $L=2$. The confidence interval is analogous to (4), with *n* substituted by $n(L-1)$.

From a Bayesian perspective, we can, as before, specify a conjugate Gamma prior for *d*, obtaining the posterior distribution

17 $$\begin{aligned} {\hat{d}}_L|\varvec{\mu }_{L} \sim Gamma \left( a+n(L-1),b+\sum _{i=1}^n\sum _{l=2}^L (l-1)\log (\mu _{i,l})\right) . \end{aligned}$$

We note that the expression in (16) is equivalent to the corrected estimator proposed by^34^ in a famous online comment. Thus, we refer to it as MG estimator. We will discuss this equivalence more in detail in “Connection with existing likelihood-based methods”. Although the availability of a closed-form expression is appealing, in “A comparison of the assumptions behind the TWO-NN, MG, and Gride” we will motivate why Gride is preferable to MG.

#### Connection with existing likelihood-based methods

Here, we discuss how our proposals are closely related to estimators introduced in the seminal work of^1^ (LB) and the subsequent comment of^34^ (MG). This relationship is not surprising, since the two estimators were derived within the same theoretical framework. Recall that we defined $\mu _{i,j,k} = r_{i,k} / r_{i,j}$. Given two integer values $q_1<q_2$, the LB estimator is defined as

18 $$\begin{aligned} \texttt {LB}(q_1,q_2) = \frac{1}{q_2-q_1+1}\sum _{k=q_1}^{q_2}{\hat{m}}_k,\quad \quad {\hat{m}}_k = \frac{k-1}{n}\sum _{i=1}^n \left( \sum _{j=1}^{k-1}\log \left( \mu _{i,j,k}\right) \right) ^{-1}, \end{aligned}$$

where we exploit the equality $\sum _{l=2}^L (l-1) \log (\mu _{i,1,l}) = \sum _{l=1}^{L-1} \log (r_{i,L}/r_{i,l})$ to re-express their estimators in terms of the $\mu$’s. The estimator proposed in^34^ considers a different expression for ${\hat{m}}_k$, that we denote by ${\hat{m}}'_k$:

19 $$\begin{aligned} {\hat{m}}'_k = \frac{n(k-1)}{\sum _{i=1}^n \left( \sum _{j=1}^{k-1}\log \left( \mu _{i,j,k}\right) \right) }. \end{aligned}$$

The LB estimator combines the terms contributing to the likelihood through a simple average. These estimators are evaluated for different values of the larger NN order, considered between $q_1$ and $q_2$, and then averaged together again. MacKay and Ghahramani^34^ noted that the authors should have instead averaged the inverse of the contributions to be coherent with the proposed theoretical framework. This correction leads to the expression in (19), which is equivalent to the MLE for MG, as stated in Equation (16). Although the expressions are the same, we believe that our derivation presents an advantage. Indeed, starting from the distributions of the ratios of NNs distances, we can effortlessly derive uncertainty quantification estimates, as in (4), by simply exploiting well-known properties of the Pareto distribution.

Following the LB strategy, one can pool together different estimates obtained with MG over a set of different NN orders $L\in \{L_1;\,\ldots ;\,L_2\}$ by considering the value $\sum _{l=L_1}^{L_2}{\hat{m}}'_l/(L_2-L_1+1)$. Unless otherwise stated, when computing the MG estimator in “Results” we will adopt this averaging approach, as implemented in the R package Rdimtools^37^.

Among all the discussed estimators, Gride is the genuinely novel contribution of this work, and it is also the most general and versatile. Indeed, it relies on a single ratio of distances for each data point (similarly to the TWO-NN) while considering information collected on larger neighbors (similarly to MG) and, therefore, is likely to be more compliant with the independence assumption.

#### A comparison of the assumptions behind the TWO-NN, MG, and Gride

We now discuss the similarities and differences among the three estimators presented so far.

The first point we need to make is that, similarly to Theorem 2.1, Theorems 2.2 and 2.3 can be proved only assuming $\rho$ to be constant. However, from a practical perspective, the novel estimators are empirically valid as long as the density $\rho$ is approximately constant on the scale defined by the distance of the *L*-th NN $r_{i,L}$ (MG) and the $n_2$-th NN $r_{i,n_2}$ (Gride), respectively. Again, we will refer to this assumption as local homogeneity. In the following, when we need to underline the dependence of the introduced families of estimators on specified NN orders, we will write $\texttt {MG}(L)$ and $\texttt {Gride}(n_1,n_2)$.

Both MG and Gride extend the TWO-NN rationale, estimating the $\texttt {id }$ on broader neighborhoods. By considering the ratio of two NNs of generic order, Gride extracts more information regarding the topology of the data configuration. Moreover, monitoring how Gride’s estimates vary for different NNs orders allows the investigation of the relationship between the dataset’s $\texttt {id }$ and the scale of the neighborhood. That way, it is possible to escape the strict, extremely local point of view of the TWON-NN. This property reduces the distortion produced by noisy observations in estimating the $\texttt {id }$.

With its alternative formulation, MG reaches a similar goal exploiting the properties of all the consecutive ratios up to the highest NNs order that we consider. MG is appealing, being an intuitive extension of the TWO-NN model, and possessing a closed-form expression for its MLE and confidence interval.

**Figure 2:**
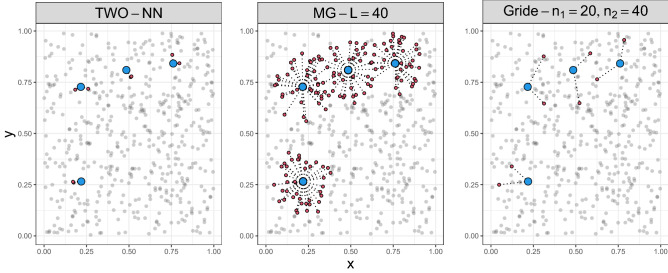
Neighboring points (in red) and distances (dotted lines) involved in the $\texttt {id }$ estimation centered in four data points (in blue). Each panel corresponds to one model: TWO-NN, MG$(L=40)$, and Gride$(n_1=20,n_2=40)$, respectively.

However, we are going to show that Gride is more reliable when it comes to real datasets. To support this statement, we need to discuss the validity of the assumptions required for deriving these estimators. As mentioned in “Likelihood-based TWO-NN estimators”, the main modeling assumptions are two: the local homogeneity of the underlying Poisson point process density and the independence among ratios of distances centered in different data points. These assumptions affect the three estimators differently. To provide visual intuition, in Fig. 2 we display 500 points generated from a bidimensional Uniform distribution over the unit square. Then, we randomly select four points (in blue) and highlight (in red) the NNs involved in the computation of the ratios that are used by the TWO-NN, MG, and Gride models. We consider $\texttt {MG}(40)$, and $\texttt {Gride}(20,40)$.

For both $\texttt {MG}(L)$ and $\texttt {Gride}(n_1,n_2)$, the local homogeneity hypothesis has to hold for larger neighborhoods, up to the NN of orders $L>2$ and $n_2>2$, respectively. We will empirically show that while $\texttt {MG}$ and $\texttt {Gride}$ are more reliable than TWO-NN if used on dense configurations, care should be taken when interpreting the results obtained from scarce datasets. Although the stricter local homogeneity assumption affects the two estimators similarly, they are not equally impacted by the assumption of independence of the ratios. By comparing the second and third panels of Fig. 2, we observe that MG, in its computation, needs to take into account all the distances among points and its NNs up to the *L*-th order. When *L* is large and the sample size is limited, neighborhoods centered in different data points may overlap, inducing dependence across the ratios and violating one of our fundamental assumptions. Gride instead uses only two distances, and the probability of shared NNs across different data points is lower, especially if large values of $n_1$ and $n_2$ are chosen.

Given the previous points, in the experiments outlined in the next section, we set $n_2=2n_1$. Our simulation studies showed that this choice is robust to the dimensionality of the dataset and provides a good trade-off between the scalability of the algorithm and the careful assessment of the dependence of the id to the scale.

## Results

The numerical experiments carried out in this section are based on the functions implemented in the Python package DADApy^38^ (available at the GitHub repository sissa-data-science/DADApy ) and in the R package intRinsic^39^, unless otherwise stated.

### Simulation studies

#### Gride is asymptotically unbiased

**Figure 3:**
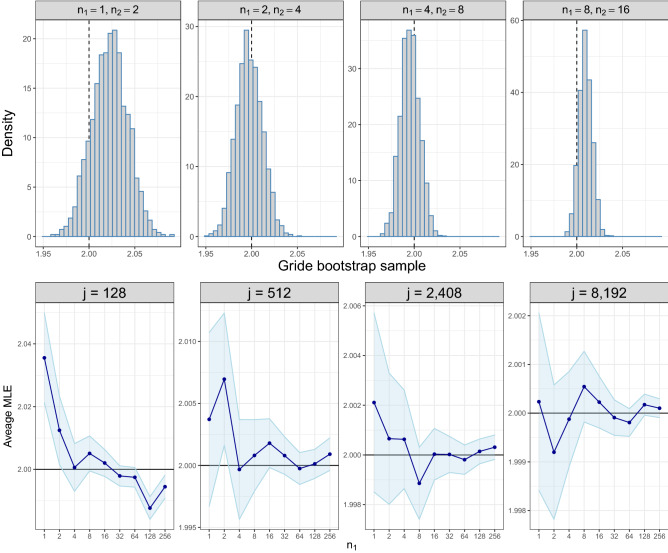
Top row: histograms of the Gride parametric bootstrap samples estimated within the frequentist framework for different NN orders. Note that the first panel corresponds to the TWO-NN model. Bottom row: average Gride MLE obtained over different NN orders. The various panels showcase the different neighborhood sizes that are considered for computing the estimates. The error bands display the $95\%$ confidence intervals on the average MLE.

First, we empirically show the consistency of Gride. This result represents an important gain with respect to the TWO-NN estimator. We sample 10000 observations from a bivariate Gaussian distribution, and aim at estimating the true $\texttt {id}=2$. To assess the variance of the numerical estimator devised from the log-likelihood in (15), we resort to a parametric bootstrap technique. We collect 5000 simulations as bootstrap samples under four different scenarios that we report in the panels displayed in the top row of Fig. 3. A similar analysis can be performed within the Bayesian setting, studying the concentration of the posterior distribution. We display the posterior simulations in the Supplementary Material (Fig. S2) . We see that, as the NNs order increases, the bootstrap samples are progressively more concentrated around the truth, with minor remaining bias due to the lack of perfect homogeneity in the data generating process.

As a second analysis, we show that high-order Gride estimates are also empirically unbiased when the homogeneity assumption of the underlying Poisson process holds. To create a dataset that complies as much as possible with the theoretical data-generating mechanism, we start by fixing a pivot point, and we generate a sequence of $n=30000$ volumes of hyperspherical shells from an exponential distribution under the homogeneous Poisson process framework. Let us denote the sequence of these volumes with $\{v_i\}_{i=1}^{n}$. Once the volumes are collected, we compute the actual distance (radius) from the pivot point by using Eq. (1) with $d=2$ and $r_0=0$. Thus, we have $r_1=\sqrt{v_1/\omega _2}\,$, $r_2=\sqrt{(v_1+v_2)/\omega _2}$, and so on. Then, we generate the position of the *i*-th point at a distance $r_i$ from the pivot by sampling its angular coordinates from a uniform distribution with support $\left[ 0, 2\pi \right)$ for each *i*.

The panels in the bottom row of Fig. 3 show the id estimates as a function the number of points closest to the pivot $j \in \{128;\, 512;\, 2048;\, 8192\}$. We employ different NN orders keeping the ratio ${n_2}/{n_1}=2$ fixed and we increase $n_1$ geometrically from 1 to 256 (*x*-axis). In this experiment, the id is estimated via MLE on 1000 repeated samples. Given the sample of 1000 estimates ${\hat{d}}$ we compute its average with its 95% confidence bands. The first three panels show a small but consistent bias for the id estimated with $n_1=1$ (TWO-NN) and $n_1=2$. The most viable explanation for the behavior of the estimator at small $n_1$ is the statistical correlation: the ${\dot{\mu }}$’s entering in the likelihood (see Eq. 10) are computed at nearby points and, as a consequence, they cannot be considered purely independent realizations. But, remarkably, this correlation effect is significantly reduced when larger values of $n_1$ are considered. Moreover, the slight bias we may observe at large NN orders is likely due to numerical error accumulation. Recall that the radii of the produced points are obtained from the sum of *l* volumes sampled from a homogeneous Poisson process. Given the data generating mechanism we used, the statistical error might compound across different stages.

#### Gride performance as the dimensionality grows

**Figure 4:**
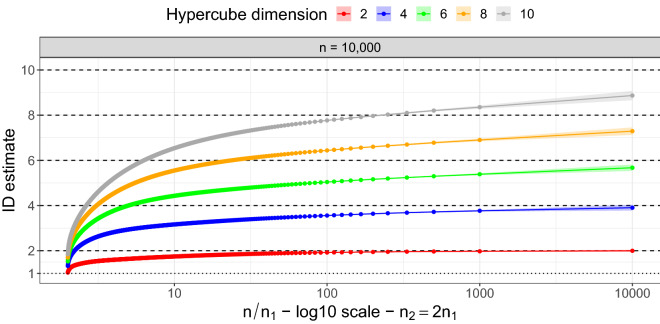
Evolution of the id estimates as a function of the ratio $n/n_1$ (logarithmic scale) computed on uniform hypercubes characterized by different sample sizes and increasing true $\texttt {id }$. Gride is computed setting $n_2=2n_1$. The horizontal lines highlight the true values of the id.

We investigate the evolution of the $\texttt {id }$ estimates produced by Gride as we vary the size of the neighborhoods considered in the estimation and the true $\texttt {id }$. To simultaneously assess the variability of our estimates, we generate 50 replicated datasets from a Uniform random variable over hypercubes in dimensions $d\in \{2;\,4;\,6;\,8;\,10\},$ with sample size $n = 10000.$ We choose to keep the sample size of this experiment relatively low (w.r.t. high $\texttt {id }$ values, such as $d=10$) to showcase the effect of the negative bias that is known to affect many id estimators in large dimensions. For each dataset, we apply a sequence of Gride models with varying NN orders, fixing $n_2=2n_1$, with $n_1 \in \{ 1;\,10;\,20;\,\ldots ;\,n/2-10\}$. We average the results over the 50 Monte Carlo (MC) replicas and plot them as functions of the ratio $n/n_1$, along with their MC standard errors (shaded areas). We display the results in Fig. 4. Note that plotting the resulting $\texttt {id }$ as a function of $n/n_1$ provides an idea of the evolution of the estimates as the considered scale goes from extended neighborhoods ($n/n_1\approx 2$) to highly local neighborhoods ($n/n_1 = n$).

Indeed, this graphical representation allows us to monitor the effect induced by the scale: the negative bias becomes more prominent as the sizes of the considered neighborhoods increase, collapsing the estimates towards 1 as $n/n_1\rightarrow 2$, as expected. Focusing on highly local neighborhoods (i.e., TWO-NN case) produces more accurate estimates on average since the underlying modeling assumptions are more likely to be met. This accuracy is achieved at the cost of high dispersion, which is mitigated by the increment of NN orders. In the Supplementary Material, we report similar results obtained using smaller sample sizes, $n\in \{500;\, 5000\}$ to assess how the uncertainty of the $\texttt {id }$ estimates changes as a function of *n* (Fig. S4).

#### Comparison of the evolution of likelihood-based id estimates in the presence of noise

We present different studies on the evolution of the estimates of the $\texttt {id }$ applied to datasets contaminated with noise, focusing on the comparison of model-based id estimators such as Gride, TWO-NN, LB, and MG. Facco et al.^2^ showed that a scale-dependent analysis of the id is essential to identify the correct number of relevant directions in noisy data. In their work, the authors proposed to subsample the dataset to increase the average distance from the second NN (and thus the average neighborhood size) involved in the TWO-NN estimate. With the same aim, we instead adopt a different approach. Again, we apply a sequence of Gride models on the *entire dataset* to explore larger regions: the higher $n_1$ and $n_2$ are, the larger is the average neighborhood size analyzed.

**Figure 5:**
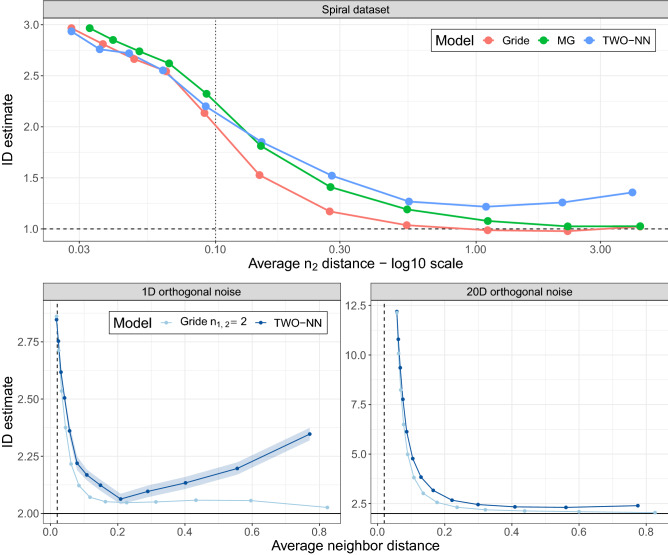
Top panel: study of the evolution of the $\texttt {id }$ on a simulated Spiral dataset. The three lines represent three different models: Gride, MG, and the decimated TWO-NN. Bottom panels: analysis of the impact of the scale on the id estimates comparing Gride models of order ratios $n_{2,1}=2$ vs. the TWO-NN estimator performed on a 2D noisy Gaussian dataset. The error bounds ($\pm 2$ std. err.) are visible only for one model.

As a first example, we focus on a second Spiral dataset generated as described in “Likelihood-based TWO-NN estimators”. We generate a sample of size $n=5000$ setting ${\bar{S}}=6$, $\sigma _x=\sigma _y=\sigma _z=0.1$. Specifically, we study the $\texttt {id }$ as a function of the size of the neighborhood by comparing three estimators: Gride with $n_2=2n_1$, MG with $L=n_2$ (single estimate, not averaged), and the decimated TWO-NN ($n_2=2$). In this simulation, we compute the estimates setting $n_1\in \{2^j\}_{j=1}^{10}$. The results are displayed in the top row of Fig. 5, where the *x*-axis reports the $\log_{10}$ average distance from the furthest nearest neighbor $n_2$ at each step. Gride plateaus around the true $\texttt {id }$ value faster than the competitors. Eventually, MG reaches a similar result, but much larger neighborhoods are required. Lastly, the decimated TWO-NN shows an $\texttt {id }$ evolution pointed in the right direction, but as the ratio of data considered decreases, its performance deteriorates.

As a second experiment devised to investigate the impact of the scale on the id estimates, we simulate 50000 data points from a two-dimensional Gaussian distribution and perturb them with orthogonal Gaussian white noise. We compare the results obtained in two cases: one-dimensional (1D) and twenty-dimensional (20D) noise; in both cases, the perturbation variance is set to $\sigma ^2 = 0.0001$. The second row of Fig. 5 reports the results of the scale analysis done with TWO-NN and Gride with $n_{2, 1} =2$. Following^2^, we apply the TWO-NN estimator on several subsets of the original data and report the average id with its 95% confidence intervals. Both in the case of high and low dimensional noise, Gride reaches the true value 2 at smaller scales than the TWO-NN estimator. The left panel also shows that the decimation protocol of TWO-NN can introduce a bias at large scales when the size of the replicates becomes small. In our experiment, by halving the sample size at each decimation step, we use subsets with 12 data points when ${\bar{r}} \approx 0.8$. At a comparable scale, Gride performs much better since it always maximizes the likelihood utilizing all of the original 50000 data points.

**Figure 6:**
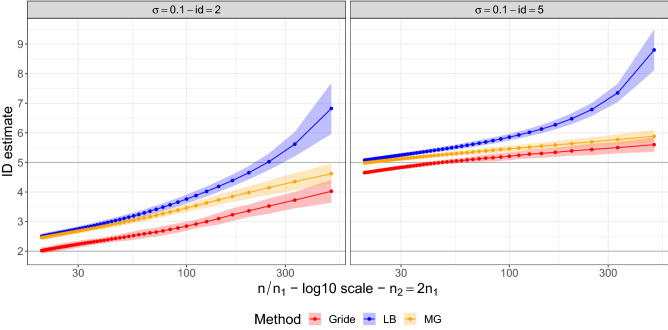
Each panel shows the average estimates of the $\texttt {id }$ over 30 replicates with three different methods: Gride (with $n_2=2n_1$), LB, and MG. Each dataset has 1000 observations. The confidence bands are drawn at $\pm 2$ standard errors. In the left panel the true $\texttt {id }$ is $d=2$, in the right panel the true $\texttt {id }$ is $d=5$.

In our last experiment on simulated data, we compare the performance of the MLEs introduced by^1^ (LB) and modified by^34^ (MG) with Gride in term of robustness to noise. To compute the first two estimators, we rely on the implementation contained in the R package Rdimtools^37^. As in the previous experiments, we want to compare how well the different estimators can escape the overestimation of the id induced by the presence of noise in the data. We have already established that Gride can exhibit a plateau around the true $\texttt {id }$ when enough signal is available (conveyed both in terms of large sample size and low level of noise). Instead, we now test our estimator in a similar but more challenging context, considering the limited sample size and the increasing noise level. Thus, we generate 30 replicas of $n\in \{1000;\,5000\}$ observations sampled from a Gaussian distribution. We consider two possible values for the intrinsic dimension: $d \in \{2;\,5\}$. Each dataset was then embedded in a $D = d+5$ dimensional space and contaminated with independent Gaussian noise $N(0,\sigma ^2)$, with $\sigma \in \{0;\,0.1;\,0.25;\,0.50\}$, expecting the random noise to induce an incremental positive bias in the id estimation. To let the estimators gather information from increasingly wider neighborhoods, we consider the relation $n_2 = 2n_1$, with $n_1=\{2,\ldots ,50\}$. The same range is considered for the averages computed with LB and MG. In the Supplementary Material, we report the plots summarizing all the results (Fig. S8). Here, we focus on the representative scenario where $n=1000$ and $\sigma =0.1$. The results are shown in Fig. 6.

From the panels in Fig. 6 we observe that the estimators present similar patterns for the two considered $\texttt {id }$ values. As expected, the id estimates are inflated by the addition of noise to the data. For small neighborhoods, Gride and MG show similar behaviors, while as $n_1$ increases MG tends to perform similarly to LB. Gride instead decreases faster than the two competitors. Thus, our proposal is more robust than the two model-based competitors when handling noisy datasets.

### Comparisons with other estimators

In the remaining of the paper, we investigate the evolution of the id estimates obtained on simulated and real benchmark datasets, comparing Gride and the TWO-NN models to three other state-of-the-art estimators: DANCo^20^, GeoMLE^30^, and ESS^31^. In our analyses, we employ both the MATLAB package of^40^ and the R package intrinsicDimension to compute DANCo, the code available at the GeoMLE GitHub repository to compute GeoMLE, and again the R package intrinsicDimension^41^ to obtain the ESS values (employed here as a global id estimator)﻿. For each model, we opted the default parameter specifications available in the code, whenever possible. Finally, let us denote the number of observations and the number of features with *n* and *D*, respectively.

#### Application to datasets with known id

**Figure 7:**
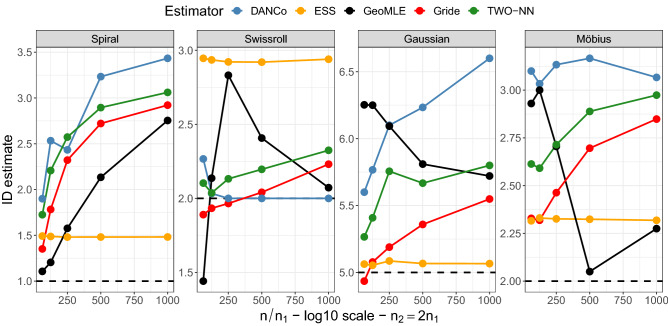
Evolution of the estimates of the id of the Spiral, Swissroll, Gaussian, and Möbius datasets computed via DANCo, GeoMLE, ESS, TWO-NN, and Gride while varying the considered neighborhood size.

To start, we employ four synthetic datasets with known id. The datasets we use are generated with (1) the Spiral transform we introduced earlier ($D=3,$ id=1), (2) the Swissroll mapping^11^ ($D=3,$ id=2), (3) a five-dimensional (id=5) Normally distributed cloud of points embedded in dimension $D=7$ , and (4) the 10-Möbius dataset^17,42^ ($D=3,$ id=2). In all datasets, we slightly perturb the original coordinates with Gaussian random values to assess if the estimators are robust to noise and to study the effect of the scale of the considered neighborhoods. We perform the estimations of the ids over 30 replications of size $n=1000$, and then we average the results. To monitor the effect of the scale, we decimate the data by considering a fraction $n/n_1$ of observations, where $n_1=\{2^j\}_{j=0}^4$. This procedure holds for all the estimators but Gride, for which we change the NN-orders. The results are summarized in Fig. 7. In the Supplementary Material, we report an additional figure containing the error bands of the estimates (Fig. S5).

All the competitors behave similarly, returning estimates that decrease as broader neighborhoods are considered, except for ESS, which remains relatively constant regardless of the dataset size, and GeoMLE. ESS performs best on the Gaussian data but tends to slightly inflate the estimates in the Swissroll case. Gride almost always outperforms the decimated TWO-NN, successfully overcoming the noise effect. That said, a uniformly better estimator does not emerge. For example, DANCo works extremely well for the Swissroll data while obtaining worse performance than its competitors in the other datasets, especially when the full datasets, with no decimation, are considered ($n/n_1=1000$). Nonetheless, we are reassured by the fact that Gride provides results that are either better or, at worse, in line with the other state-of-the-art estimators. Furthermore, an important feature of Gride appears from Fig. S5 in the Supplementary Material: its uncertainty decreases as larger neighborhoods are considered. At the same time, as decimation increases, results become more volatile for most of the competitors.

#### Application to real datasets

**Figure 8:**
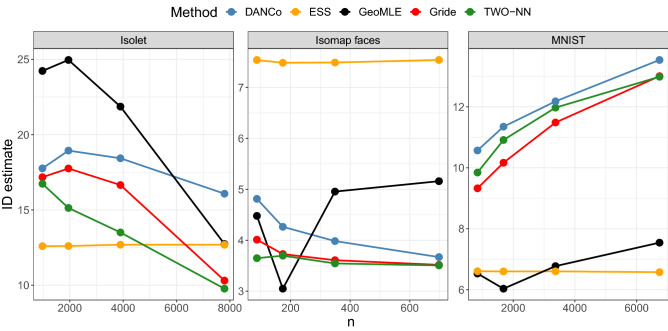
Evolution of the estimates of the id of the datasets Isolet, Isomap faces, and MNIST (digit 1) obtained with DANCo, GeoMLE, ESS, TWO-NN, and Gride varying the considered sample size.

Following^20^, we consider the MNIST (focusing on the training points representing digit 1: $n = 6742,\; D = 7797$) and the Isolet datasets ($n = 784,\; D =617$). Moreover, we consider the Isomap faces dataset ($n = 698,\; D =4096$) as in^33,43^, and the CIFAR-10 dataset as in^44^ (training data, $n = 50000,\; D =3072$).

id **estimation as a function of the sample size**. We study how the estimates returned by the five considered models change when applied to the Isolet, Isomap faces, and MNIST datasets, as we consider different sample sizes. For each dataset, we randomly extract six sub-samples of size *n*/*k*, where $k\in \{1;\,2;\,4;\,8\}$, and use them to estimate the $\texttt {id }$. To obtain more robust estimates, each sub-sample of size *n*/*k* is replicated *k* times, and the resulting estimates are subsequently averaged. We report the results in Fig. 8. First, we observe that most estimators yield heterogeneous results across the data sizes, with the only exception of ESS, which produces coherent estimates regardless of the sample size. However, in line with previous studies, the ESS estimator tends to struggle when the sample size is limited w.r.t. the number of features. Indeed, as noted in^43^, from the second panel we observe that ESS overestimates the expected value for the $\texttt {id }$ of the Isomap faces dataset. GeoMLE obtains mixed results: while producing reasonably consistent results on MNIST, it provides widely variable estimates on the remaining two datasets. Gride and TWO-NN provide results that are, overall, very close to the ones obtained with DANCo. This result is remarkable, especially when considering the high-dimensional nature of the datasets. Moreover, albeit our proposal is exclusively based on the information provided by the distances among data points (while all the competitors utilize some additional topological features), we do not observe any systematic bias or abnormal pattern in the estimates.

**Figure 9:**
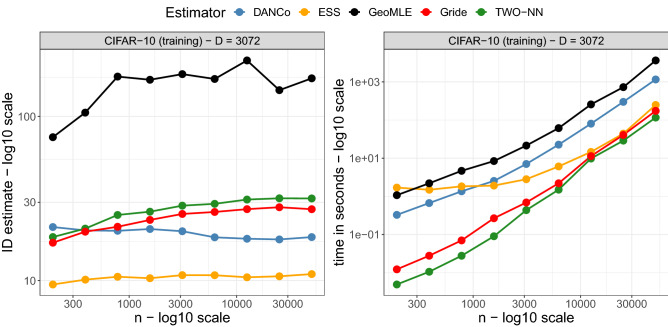
Trajectories of estimated $\texttt {id }$s (left panel) and elapsed times in seconds (right panel) obtained on the CIFAR-10 (training) dataset with DANCo, GeoMLE, ESS, TWO-NN, and Gride.

**Differences in computational costs**. Finally, we investigate the differences in computational costs for various estimators. To this end, we consider two versions of the CIFAR-10 dataset, chosen because of its high dimensionality in both the numbers of instances and features. On the one hand, to study how the different algorithms scale as the considered sample size increases, we utilize the CIFAR-10 (training) dataset. We compute the $\texttt {id }$ estimates after sub-sampling the datasets producing samples of size *n*/*k*, with $k\in \{2^j\}_{j=0}^7$, while leaving *D* unaltered and equal to 3072. The results of this experiment are shown in Fig. 9, where we display the retrieved $\texttt {id }$s and the elapsed time in seconds. On the other hand, we also explore how the algorithms scale as the number of features increases by employing a subset of the CIFAR-10 dataset, where we focus on $n=5000$ pictures of cats. These images were re-sized (both shrunk and enlarged) to $q\times q$ pictures, where *q* assumes values between 8 and 181. Notice that the datasets encode the RGB information for each picture. Therefore, the number of features is $D =3q^2$, ranging from a minimum of 192 to a maximum of 98283. We defer the results of the latter experiment to the Supplementary Material (Fig. S6). In both cases, we observe that GeoMLE presents highly varying results, especially when we consider a variable number of features. The other estimators, on the contrary, deliver consistent results, with Gride providing similar estimates to DANCo. Moreover, while the $\texttt {id }$ estimates are still on par with the competitor, we observe an important gain in computational speed: Gride is considerably faster than its competitors, and it is second only to the TWO-NN when dealing with small datasets. For example, to run the model on the complete CIFAR-10 (training) dataset, ESS takes 1.43 times the seconds needed to run Gride, DANCo 6.66 times, and GeoMLE 21 times.

## Discussion

In this paper, we introduced and developed novel distributional results concerning the homogeneous Poisson point process related to the estimate of the id, a crucial quantity for many dimensionality reduction techniques. The results extend the theoretical framework of the TWO-NN estimator. In detail, we derived closed-form density functions for the ratios of distances between a point and its nearest neighbors, ranked in increasing order.

The distributional results have a theoretical importance per se but are also useful to improve the model-based estimation of the id. Specifically, we have discussed two estimators: MG and Gride. The first one builds on the independence of the elements of the vector of consecutive ratios $\varvec{\mu }_{L}$, which we exploited to derive a closed-form estimator with lower variance than the TWO-NN. We showed how this estimator is equivalent to the one proposed in^34^. However, in real cases, considering multiple ratios of distances for each point in the sample can violate the assumed independence.

Our main proposal is Gride, an estimator based on NNs of generic order capable of mitigating the issues mentioned above. We showed that the latter estimator is also more robust to the presence of noise in the data than the other model-based methods. We remark that the inclusion of NNs of higher orders has to be accompanied by stronger assumptions on the homogeneity of the density of the data-generating process. Nonetheless, by dedicated computational experiments, we have shown that one can weaken the assumption of homogeneity of the Poisson point process. Indeed, given a specific point in the configuration, the homogeneity hypothesis should only hold up to the scale of the distance of the furthest nearest neighbor entering the estimator.

To summarize, when dealing with real data, we face a complex trade-off among the assumptions of density homogeneity, independence of the ratios, and robustness to noise. On the one hand, the TWO-NN is more likely to respect the local homogeneity hypothesis but is extremely sensitive to measurement noise since it only involves a narrow neighborhood of each point. On the other hand, MG focuses on broader neighborhoods, which makes it more robust to noisy data. However, their definition also imposes a more substantial local homogeneity requirement. It is also more likely to induce dependencies among different sequences of ratios. We believe that Gride provides a reliable alternative to the previous two maximum likelihood estimators, being both robust to noise and more likely to comply with the independence assumptions.

Moreover, we have also compared Gride with other state-of-the-art methodologies, such as DANCo, ESS, and GeoMLE, over various simulated and well-known benchmark datasets. We have observed that Gride obtained performance on par with its competitors in terms of id estimation, especially similar to DANCo. This fact is even more remarkable if we consider that, differently from the competitors, our estimator is exclusively based on the information extracted from the distances among data points. Therefore, Gride represents a valuable tool, primarily because of its simplicity and computational efficiency.

The results in this paper pave the way for many other possible research avenues. First, we have implicitly assumed the existence of a single manifold of constant id. However, it is reasonable to expect that a complex dataset can be characterized by multiple latent manifolds with heterogeneous $\texttt {id }$s. Allegra et al.^45^ extended the TWO-NN model in this direction by proposing Hidalgo, a tailored mixture of Pareto distributions to partition the data points into clusters driven by different $\texttt {id }$ values. It would be interesting to combine the Hidalgo modeling framework with our results, where the distribution in Eq. (10) can replace the Pareto mixture kernels. Second, the estimators derived from the models do not directly consider any source of error in the observed sample. Although we showed how one could reduce the bias generated by this shortcoming by considering higher-order nearest neighbors that allow escaping the local distortions, we are still investigating how to address this issue more broadly. For example, a simple solution would be to model the measurement errors at the level of the ratios, accounting for a Gaussian noise that can distort each $\mu _i$. By focusing directly on the distribution of the distances among data points in an ideal, theoretical setting, we can obtain informative insights on how to best model the measurement noise.

## Supplementary Information

Supplementary Information.

## Data Availability

The script to generate and analyze the datasets discussed in the current study are reported in the GRIDE_repo GitHub repository, available at https://github.com/Fradenti/GRIDE_repo. The real datasets utilized in the manuscript are openly available online at the following links: Isolet, Isomap, MNIST, and CIFAR-10.
