# Supplementary material for: The generalized ratios intrinsic dimension estimator
Source: Sci Rep. 2022 Nov 21;12:20005. doi: 10.1038/s41598-022-20991-1 (PMC9678878; doi:10.1038/s41598-022-20991-1)
Supplement: Supplementary file 1 — Supplementary Information. [file 41598_2022_20991_MOESM1_ESM.pdf]

Supplementary Material for "The generalized ratios intrinsic dimension estimator"

Francesco Denti[1,*], **Diego Doimo**[2], **Alessandro Laio**[2,3], **and Antonietta Mira**[4,5,†]

[*]francesco.denti@unicatt.it
[†]antonietta.mira@usi.ch
[1]Department of Statistics, Università Cattolica del Sacro Cuore, Milan, Italy
[2]SISSA, Via Bonomea 265, Trieste, Italy
[3]ICTP Strada Costiera 11, 34151 Trieste, Italy
[4]Faculty of Economics, Università della Svizzera italiana, Lugano, Switzerland
[5]University of Insubria, Varese, Italy

ABSTRACT

The supplementary material is structured as follows. Section 1 contains the proofs of the theoretical results presented in the main article, and Section 2 delineates more theoretical considerations regarding the `TWO-NN` model. Section 3 presents some other comments and theoretical results. Section 4 contains additional plots that are mentioned in the main article, while Section 5 shows the results of further simulation studies.

1 Proofs

1.1 Alternative proof of Theorem 2.1[1]

First, we remind that $(*)$ if $X \sim Exp(\rho)$ and $Y \sim Erlang(n,\rho)$ such that $X \perp\!\!\!\perp Y$, then $Z = \frac{X}{Y} + 1 \sim Pareto(1,n)$. Moreover, we can prove the following Lemma.

Lemma 1.1 (Scaling property of the Pareto distribution)**.** $X \sim Pareto(1,\alpha) \iff Y = X^q \sim Pareto(1,\alpha/q)$.

Proof. If $X \sim Pareto(1,\alpha)$, then $f_X(x) = \alpha x^{-(1+\alpha)}$. We consider the transformation $X = Y^{1/q}$ and compute $\frac{d}{dy}y^{1/q} = \frac{1}{q}y^{1/q-1}$. Then the density of Y can be expressed as:

$$f_Y(y) = \alpha y^{-(1/q+\alpha/q)}\frac{1}{q}y^{1/q-1} = \left(\frac{\alpha}{q}\right)y^{(-\alpha/q+1)}$$

which is the density of a $Pareto(1,\alpha/q)$ random variable. The converse can be shown by simply applying the inverse transformation. □

We are now ready to prove Theorem 2.1.

Proof. Let us consider a generic point $\boldsymbol{x}_i$ and its corresponding volumes $\{v_{i,l}\}_{l=1}^{n-1}$ as defined in (S.2). If the density of the Poisson point process is constant, then $v_{i,l} \overset{i.i.d.}{\sim} Exp(\rho)$ for all l. Recall that the Exponential distribution is equivalent to an $Erlang(1,\rho)$ distribution. Then, according to $(*)$, $\frac{v_{i,2}}{v_{i,1}} + 1 \sim Pareto(1,1)$. Also, we have that $\frac{v_{i,2}}{v_{i,1}} + 1 = r^d_{i,2}/r^d_{i,1}$. We can then conclude that

$$\mu_i = \frac{r_{i,2}}{r_{i,1}} = \left(\frac{v_{i,2}}{v_{i,1}} + 1\right)^{1/d},$$

which according to the previous Lemma implies $\mu_i \sim Pareto(1,d)$. □

1.2 Proof of Theorem 2.2

Proof. The marginal distributions stated in Equation (9) follow by the application of elementary properties of Exponential, Gamma and Pareto random variables to Equation (8).

We now prove that the joint independence of the elements of the vector $\boldsymbol{\mu}_{i,L}$. We drop the observational index i for ease of exposition. Let us denote $\gamma_l = \log\left(\frac{r_l}{r_{l-1}}\right)$, for $l = 2,3,\dots,L$. We want to derive the joint density of $\boldsymbol{\gamma}_L = (\gamma_2,\dots,\gamma_L)$. To do

so, we start from the joint density of $(v_1, v_2, v_3, \dots, v_L)$, denoted by $f(v_1, v_2, v_3, \dots, v_L) = \rho^L \exp\left[-\rho \sum_{l=1}^{L} v_l\right]$. Consider the following one-to-one transformation of the vector $\boldsymbol{\gamma}_L$:

$$\gamma_1 = v_1 \quad \text{and} \quad \gamma_l = \frac{1}{d}\log\left(1 + \frac{v_l}{\sum_{k=1}^{l-1} v_k}\right),\ l = 2, \dots, L$$

with inverse

$$v_1 = \gamma_1 \quad \text{and} \quad v_l = \gamma_1 \exp\left(d\sum_{k=2}^{l-1}\gamma_k\right)(\exp(d\gamma_l) - 1),\ l = 2, \dots, L.$$

The determinant of the Jacobian matrix J associated with this transformation is
$|J| = \gamma_1^{L-1} d^{L-1} \prod_{l=2}^{L} \exp[d \cdot (L-l+1)\gamma_l]$.
Consequently, the density of the transformed vector is

$$f(\boldsymbol{\gamma}) = \rho^L \gamma_1^{L-1} d^{L-1} \exp\left[-\rho\gamma_1 \exp\left(d\sum_{l=2}^{L}\gamma_l\right)\right] \prod_{l=2}^{L} \exp[d \cdot (L-l+1)\gamma_l].$$

We then integrate out γ_1 to obtain:

$$f(\boldsymbol{\gamma}_L) = d^{L-1}\prod_{l=2}^{L}(l-1)\exp[-(l-1)d\gamma_l] = \prod_{l=2}^{L}(l-1)d\exp[-(l-1)d\gamma_l].$$

Since $f(\boldsymbol{\gamma}_L) = \prod_{l=2}^{L} f(\gamma_l)$, we can conclude that $\gamma_2, \dots, \gamma_L$ are independent exponential random variables. Finally, given that $X \sim Pareto(1,a) \iff \log(X) \sim Exp(a)$, we consider $\boldsymbol{\mu}_L = \exp(\boldsymbol{\gamma}_L)$ and conclude the proof. □

1.3 Proof of Theorem 2.3

Proof. Let $\{W_i\}_{i=1}^{n}$, $n \geq 2$, denote a sequence of independent Exponential random variables with pairwise distinct parameters λ_i. The sum of n random variables $W_i \sim Exp(\lambda_i)$ is said to follow an hypo-exponential distribution, with density

$$f_{\sum_{i=1}^{n} W_i}(w) = \left[\prod_{i=1}^{n}\lambda_i\right]\sum_{j=1}^{n}\frac{e^{-\lambda_j w}}{\prod_{\substack{l\neq j\\ l=1}}^{n}(\lambda_l - \lambda_j)}, \quad w > 0.$$

Our goal is to characterize the distribution of $\dot{\mu} = \mu_{i,n_1,n_2} = \frac{r_{n_2}}{r_{n_1}}$, with $n_2 > n_1$ integer values. First, we notice that $\dot{\mu}$ can be rewritten as telescopic product of $n_2 - n_1$ ratios, all independent and Pareto distributed:

$$\dot{\mu} = \frac{r_{n_2}}{r_{n_1}} = \frac{r_{n_2}}{r_{n_2-1}} \cdot \frac{r_{n_2-1}}{r_{n_2-2}} \cdots \frac{r_{n_1+1}}{r_{n_1}}.$$

Define $\gamma_l = \log\left(\frac{r_l}{r_{l-1}}\right)$ and consider $Y = \log(\dot{\mu})$. Then, we can write $Y = \log(\dot{\mu}) = \log\left(\frac{r_{n_2}}{r_{n_1}}\right) = \sum_{l=n_1+1}^{n_2}\gamma_l$. Since each γ_l is defined as the logarithm of a Pareto distribution, we have just shown that Y is a sum of $L = n_2 - n_1$ independent Exponential random variables with parameters ranging from $n_1 d$ to $(n_2 - 1)d$. Plugging these parameters into the the definition of hypo-exponential density, we can write the distribution of Y as

$$f_Y(y) = d\frac{(n_2-1)!}{(n_1-1)!}\sum_{j=1}^{n_2-n_1}\frac{e^{-(n_1+j-1)dy}}{\prod_{\substack{l\neq j\\ l=1}}^{n_2-n_1}(l-j)}, \quad y > 0. \tag{S.1}$$

From here, we derive the distribution for $\dot{\mu} = \exp(Y)$, transforming the last density in (S.1).

$$\begin{aligned} f_{\dot{\mu}}(\dot{\mu}) =& d\frac{(n_2-1)!}{(n_1-1)!}\frac{1}{\dot{\mu}}\sum_{j=1}^{n_2-n_1}\frac{e^{-(n_1+j-1)d\log\dot{\mu}}}{\prod_{\substack{l\neq j\\ l=1}}^{n_2-n_1}(l-j)} = d\frac{(n_2-1)!}{(n_1-1)!}\sum_{j=1}^{n_2-n_1}\frac{\dot{\mu}^{-(n_1+j-1)d-1}}{\prod_{\substack{l\neq j\\ l=1}}^{n_2-n_1}(l-j)} \\ =& d\frac{(n_2-1)!}{(n_1-1)!}\sum_{j=1}^{n_2-n_1}\frac{\dot{\mu}^{-(n_1+j-1)d-1}}{(j-1)!(n_2-n_1-j)!(-1)^{j-1}} \\ =& d\frac{(n_2-1)!}{(n_1-1)!}\sum_{k=1}^{n_2-n_1}\frac{\dot{\mu}^{-(n_2-k)d-1}}{(k-1)!(n_2-n_1-k)!(-1)^{n_2-n_1-k}} \end{aligned}$$

$$
\begin{aligned}
&= \frac{d}{\dot{\mu}^{n_2 d+1}} \frac{(n_2-1)!}{(n_1-1)!} \sum_{k=1}^{n_2-n_1} \frac{\dot{\mu}^{kd}(-1)^{n_2-n_1-k}}{(k-1)!(n_2-n_1-k)!} \\
&= \frac{d}{\dot{\mu}^{n_2 d+1}} \frac{(n_2-1)!}{(n_1-1)!} \frac{(n_2-n_1-1)!}{(n_2-n_1-1)!} \sum_{k=1}^{n_2-n_1} \frac{\dot{\mu}^{kd}(-1)^{n_2-n_1-k}}{(k-1)!(n_2-n_1-k)!} \\
&= \frac{d}{\dot{\mu}^{n_2 d+1}} \frac{(n_2-1)!}{(n_1-1)!} \frac{1}{(n_2-n_1-1)!} \sum_{l=0}^{n_2-n_1-1} \binom{n_2-n_1-1}{l} (\dot{\mu}^d)^{l+1} (-1)^{n_2-n_1-l-1} \\
&= \frac{d}{\dot{\mu}^{(n_2-1)d+1}} \frac{(n_2-1)!}{(n_1-1)!} \frac{(\dot{\mu}^d-1)^{n_2-n_1-1}}{(n_2-n_1-1)!} = (n_2-n_1)\binom{n_2-1}{n_1-1} \frac{d(\dot{\mu}^d-1)^{n_2-n_1-1}}{\dot{\mu}^{(n_2-1)d+1}} \\
&= \frac{d(\dot{\mu}^d-1)^{n_2-n_1-1} \cdot \dot{\mu}^{-(n_2-1)d-1}}{B(n_2-n_1,n_1)}, \quad \dot{\mu} > 1.
\end{aligned}
$$

In the previous derivation, we applied the following equality at the second line: $\prod_{\substack{l=1 \\ l\neq j}}^{n_2-n_1} (l-j) = (j-1)!(n_2-n_1-j)!(-1)^{j+1}$. Moreover, at the fourth line we applied the reflection property of the indexes of a sum: $\sum_{k=1}^{K} a_k = \sum_{k=1}^{K} a_{K-k+1}$. At the sixth line, we applied the Newton binomial formula. Interestingly, we can define $Z = \dot{\mu}^d - 1$ to find that $Z \sim \beta'(n_2-n_1,n_1)$, where β' denotes the *Beta prime* distribution. This property helps to find the expression for the generic moment of $\dot{\mu}$:

$$
\mathbb{E}\left[\dot{\mu}^k\right] = \mathbb{E}\left[(Z+1)^{k/d}\right] = \int_0^{+\infty} (z+1)^{1/d} \frac{z^{n_2-n_1}(1+z)^{-n_2}}{B(n_2-n_1,n_1)} dz = \frac{B(n_2-n_1,n_1-k/d)}{B(n_2-n_1,n_1)},
$$

that is well defined for $k < dn_1$. □

2 Additional details about the TWO-NN modeling background

The volumes of the hyperspeherical shells are the multivariate counterpart of the univariate inter-arrival times.
It is worth noticing that in the univariate case each $v_{i,l}$ simplifies into the distance $\Delta(\boldsymbol{x}_i, \boldsymbol{x}_{(i,l)})$ and it is called *inter-arrival time*. If the underlying Poisson point process is *homogeneous*, implying that $\rho(\boldsymbol{x}) = \rho \ \forall \boldsymbol{x}$, all the $v_{i,l}$'s are independent and identically distributed as an Exponential random variable, with rate parameter equal to the density ρ[2]. Building on the work of[3],[1] have extended this result to the multivariate case, where hyper-spherical shells defined as

$$
v_{i,l} = \omega_d \left(r_{i,l}^d - r_{i,l-1}^d \right), \qquad \text{for } l = 1,\ldots,n-1, \text{ and } i = 1,\ldots,n, \tag{S.2}
$$

are the proper multivariate extension of the univariate inter-arrival times. Therefore, as in the univariate case, we have $v_{i,l} \sim Exp(\rho)$, for $l = 1,\ldots,n-1$, and $i = 1,\ldots,n$.

Some implications of Theorem 2.1.
Using only basic properties of the homogeneous Poisson point process,[1] showed that the ratio of the distances between a point and, respectively, its second and first NNs is Pareto distributed, with scale parameter equal to 1 and shape parameter d. Within this modeling framework, the latter parameter corresponds to the id of the dataset. Recall that if $Y \sim Pareto(a,b)$ then the density function of Y is defined as $f_Y(y) = ab^a y^{-a-1}$, with $y \in (b,+\infty)$.
The most important implication of Theorem 1 is that, once a proper distance is computed among the observations we can summarize all the information contained in the data about the id with the summary statistics given by $\boldsymbol{\mu} = \{\mu_i\}_{i=1}^n$, regardless the number of features D present in a dataset $\boldsymbol{X}$. This reduces the task of id estimation into a simple, scalable, and univariate estimation problem. A detailed proof of Theorem 2.1 is contained in[4] or in Section 1.1 of this document.

The assumptions behind the TWO-NN modeling background.
As previously remarked, from a practical perspective we require that the density of the Poisson point process generating the data has to be locally constant, at least on the scale of the second NN of each point. In real applications, this hypothesis is satisfied if the available sample size is large enough, implying a densely populated space. However, this assumption may fail in regions of the support where the data points are scarce.
This issue is also linked to the *curse of dimensionality* (CoD). The sample size needed to produce a configuration of points that uniformly populates the manifold into consideration needs to scale exponentially with its dimension. To see how the CoD can affect the estimation, consider the theoretical setting in which we deal with a homogeneous Poisson process. The effect of the CoD becomes evident if we focus on the expected value and the variance of the random variable μ_i, given by

$\mathbb{E}[\mu_i] = d/(d-1)$ and $\mathbb{V}[\mu_i] = d/((d-1)^2(d-2))$, respectively. If $d \to +\infty$, then the Pareto distribution collapses to a point mass in 1. Intuitively, when the dimensionality of the space that embeds the sample diverges, the distance among data points grows. As both the numerators and the denominators of all the elements in $\boldsymbol{\mu}$ scale with the same speed, they are asymptotically indistinguishable. Therefore, in large dimensions, the hypothesis of local homogeneity is more likely to be violated when dealing with a fixed sample size. In those cases, the estimates based on the previous results are to be considered as lower bounds of the true d[5].
The assumption of *independence* among the elements of $\boldsymbol{\mu}$ allows the derivation of simple estimators for the parameter of interest. However, this is not always satisfied in practice because multiple observations can share the same NNs, and therefore the same distances. A possible solution would be to decimate the sample and eliminate the NNs shared by multiple points before the analysis. However, as already shown in[6], the estimates using the decimated samples do not substantially deviate from the ones obtained using all the data.

3 Additional details about our findings

3.1 A remark about the result of Theorem 2.2

In the main text, we discussed how Theorem 2.2 can be exploited to devised an estimator that is based on the distribution of the elements of the random vector reported in (9), devising a multivariate model. Alternatively, one can go back to the univariate modeling case by considering the transformation $\gamma_{i,l} = \log(\mu_{i,l})$, obtaining that $\boldsymbol{\gamma}_{i,l} \sim Exp((l-1)d)$ and define

$$\Gamma_{i,L} = \sum_{l=2}^{L} (l-1) \cdot \gamma_{i,l} \sim Erlang(L-1, d), \quad i = 1, \dots, n. \tag{S.3}$$

It can be proven that the MLE obtained from (S.3) is identical to the one presented in Equation (16). We name the estimators derived from Theorem 2.2 the Consecutive Ratios `id` Estimators. We remark that many other distributions can be employed using the properties of the Exponential random variables. As an example, for a generic observation i and a generic ratio of order l, the following statements are equivalent to (S.3):

$$\gamma_{i,l}^2 \sim Weibull\left(\frac{1}{2}, \frac{1}{(l-1)^2 d^2}\right), \quad \mu - \sigma \log\left((l-1)d\gamma_{i,l}\right) \sim GEV(\mu, \sigma, 0),$$

where GEV indicates the Generalized Extreme Values distribution[7]. These distribution are well known in Extreme Value Theory (EVT). Other authors have recently developed an `id` estimator in an EVT framework[8,9]: we leave the investigation of potential connections among these two fields for future research.

3.2 Distribution of the generic vector of distances

Theorem. Consider a distance Δ taking values in $\mathbb{R}^+$ defined among the data points $\{\boldsymbol{x}_i\}_{i=1}^n$, which are a realization of a Poisson point process with constant density ρ. Let $r_{i,l}$ be the value of this distance between observation i and its l-th NN. Then, the joint distribution of the the vector $(r_{i,1}, \dots, r_{i,L})$ is given by

$$f(r_{i,1}, \dots, r_{i,L}) = (\rho\omega_d d)^L \left(\prod_{l=1}^{L} r_{i,l}^{d-1}\right) \exp\left[-\rho\omega_d r_{i,L}^d\right], \tag{S.4}$$

with $r_{i,l} \in \mathbb{R}^+$, and the constraint that $r_{i,1} < r_{i,2} < \dots < r_{i,L}$. Moreover, the marginal random distance between a point $\boldsymbol{x}_i$ and its L-th NN has density

$$f(r_{i,L}) = \exp\left[-\rho\omega_d r_{i,L}^d\right] (\rho\omega_d d)^L \frac{r_{i,L}^{Ld-1}}{(L-1)! d^{L-1}}. \tag{S.5}$$

This result implies that, for $i = 1, \dots, n$, $r_{i,L} \sim GenGamma(p, a, q)$, i.e., it follows a Generalized Gamma distribution with parameters $p = d$, $a = 1/\sqrt[d]{\rho\omega_d}$, and $q = Ld$.

Proof. To simplify the notation, let us drop the subscript i. Recall that we were able to prove that $f(v_1, \dots, v_L) = \prod_{l=1}^{L} f(v_l)$, where $f(v_l) = \rho\exp(-\rho v_l)$, meaning that $v_l \overset{i.i.d.}{\sim} Exp(\rho)$. We consider the following one-to-one transformation for $l = 1, \dots, L$:

$$r_l = \left(\frac{\sum_{k=1}^{l} v_k}{\omega_d}\right)^{1/d} \iff v_l = \omega_d\left(r_l^d - r_{l-1}^d\right).$$

The determinant of the Jacobian of this transformation is $|J| = (\omega_d d)^L \prod_{l=1}^{L} r_l^{d-1}$. Thus, the distribution of the first L distances has density:

$$f(r_1,\ldots,r_L) = (\rho\,\omega_d d)^L \left(\prod_{l=1}^{L} r_l^{d-1}\right) \exp\left[-\rho\,\omega_d r_L^d\right],$$

with $r_l \in \mathbb{R}^+$ and the constraint that $r_1 < r_2 < \ldots < r_L$.
We can also derive the marginal distribution of the generic distance r_L. This can be easily done by repeatedly integrating out the smallest distance r_l over $(0, r_{l+1})$, $l = 1,\ldots,L-1$. In formulas:

$$\begin{aligned} f(r_L) &= (\rho\,\omega_d d)^L r_L^{d-1} \exp\left[-\rho\,\omega_d r_L^d\right] \int_0^{r_L} \int_0^{r_{L-1}} \cdots \int_0^{r_2} \left(\prod_{l=1}^{L-1} s_l^{d-1}\right) ds_1 \cdots ds_{L-1} \\ &= \exp\left[-\rho\,\omega_d r_L^d\right] (\rho\,\omega_d d)^L \frac{r_L^{Ld-1}}{(L-1)!\,d^{L-1}}. \end{aligned}$$

We conclude that the generic distance from a point to its L-th NN follows a Generalized Gamma distribution, whose density is given by

$$f(x) = \frac{p/a^q}{\Gamma(q/p)} x^{q-1} e^{-(x/a)^p}, \qquad x, a, p, q > 0.$$

Therefore, $f(r_L)$ is a Generalized Gamma density with parameters $p = d$, $\quad a = \frac{1}{\sqrt[d]{\rho\,\omega_d}}$, $\quad q = Ld$. There is another, faster way to recover this last result. Since $v_l \sim Exp(\rho)$ for each $l = 1,\ldots,L$, it is easy to see that the volume of the hyper-sphere of radius r_L, defined as $V_L = \sum_{l=1}^{L} v_l = \omega_d r_L^d$ follows an Erlang distribution: $V_L \sim Gamma(L,\rho)$. Then,

$$V_L \sim Gamma(L,\rho) \iff r_L^d = \frac{V_L}{\omega_d} \sim Gamma(L, \omega_d \rho) \iff r_L \sim GenGamma\left(d, \frac{1}{\sqrt[d]{\rho\,\omega_d}}, Ld\right).$$

□

4 Additional figures

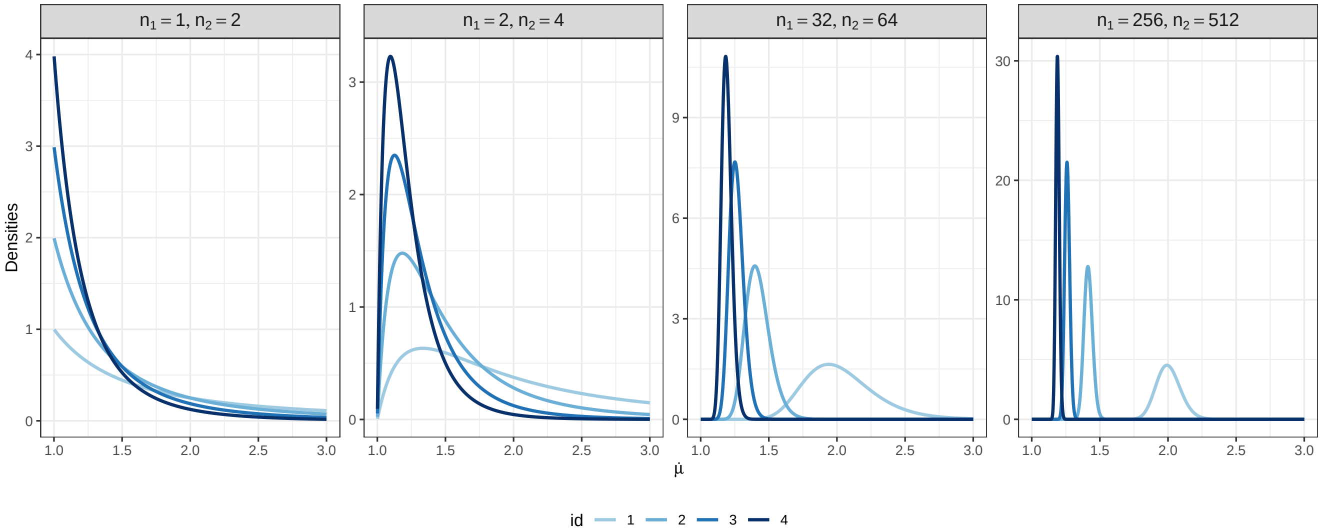

Figure S1. Examples of density functions characterizing the random variable $\dot{\mu}$ as defined in Equation (10). The different colors correspond to different values of the `id` parameter d, while the panels display the various order of NNs considered for the ratios.

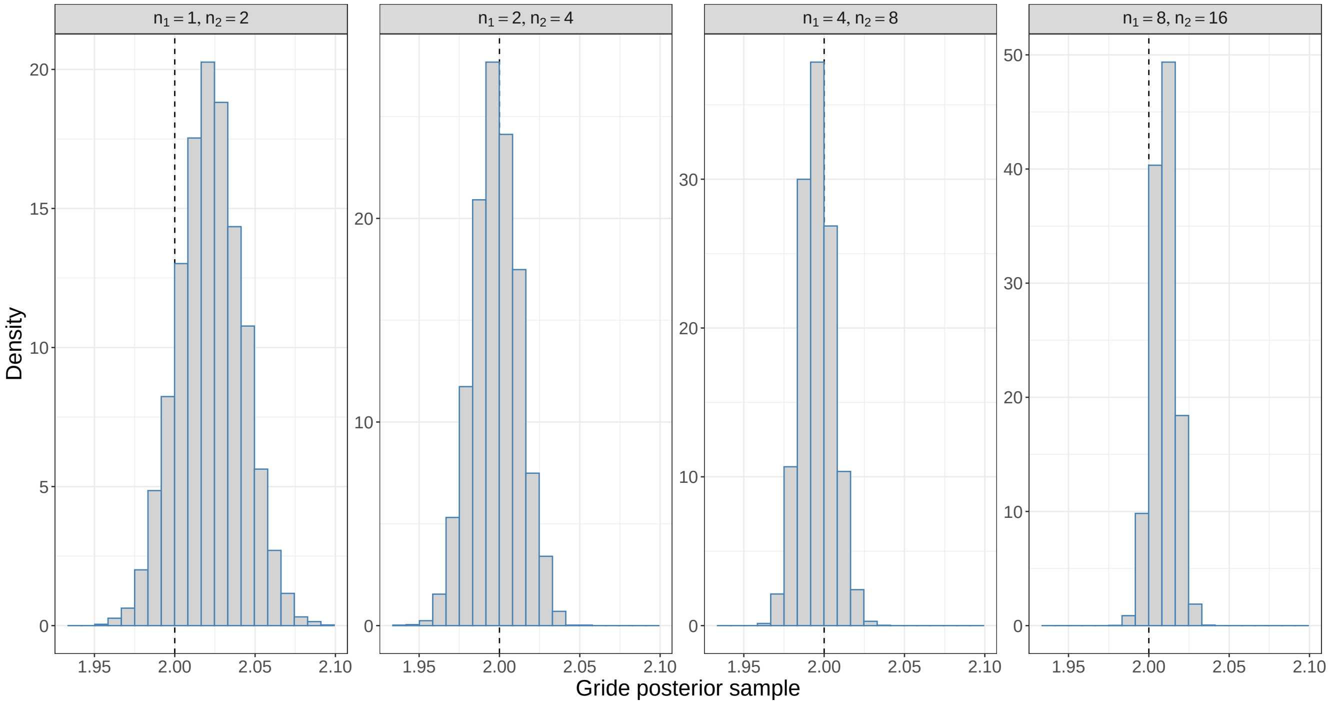

Figure S2. Histograms of the posterior samples for the `Gride` models estimated within the Bayesian framework. The first panel corresponds to the `TWO-NN` model.

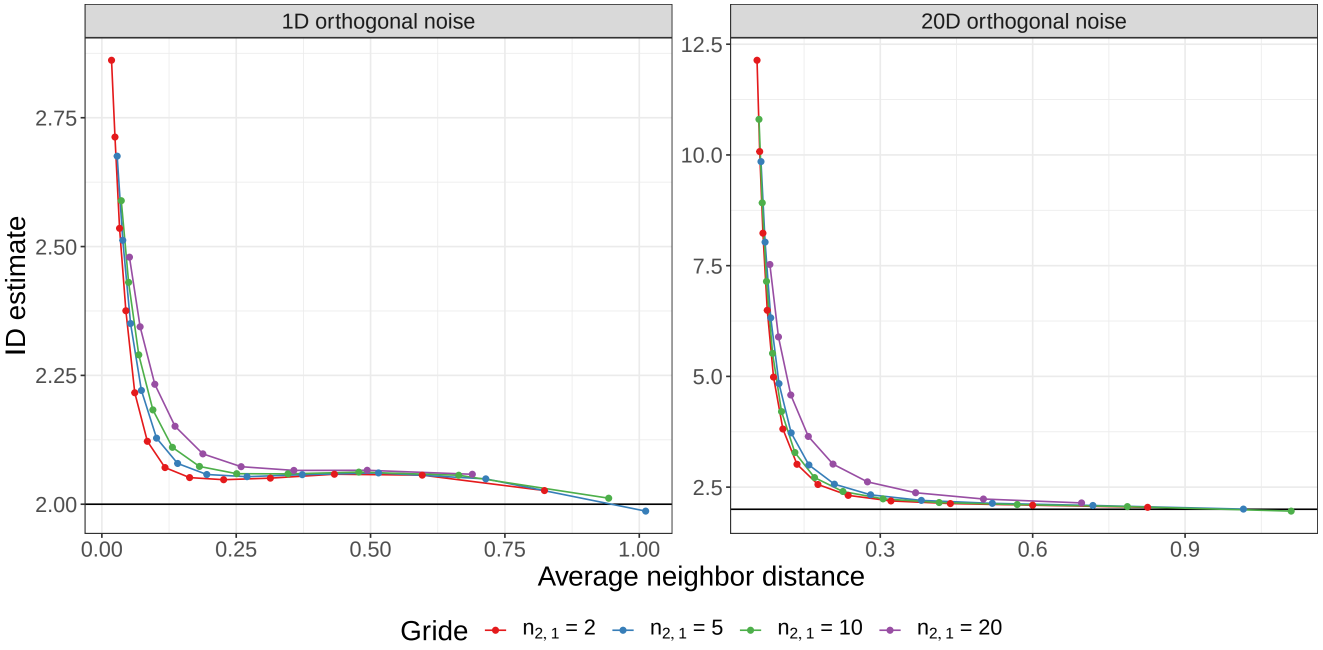

Figure S3. Analysis of the impact of the scale on the `id` estimates for different `Gride` models performed on a 2D noisy Gaussian dataset. The `id` is calculated maximizing the likelihood of Equation (10); the error bars computed with the Fisher information are smaller than the marker size.

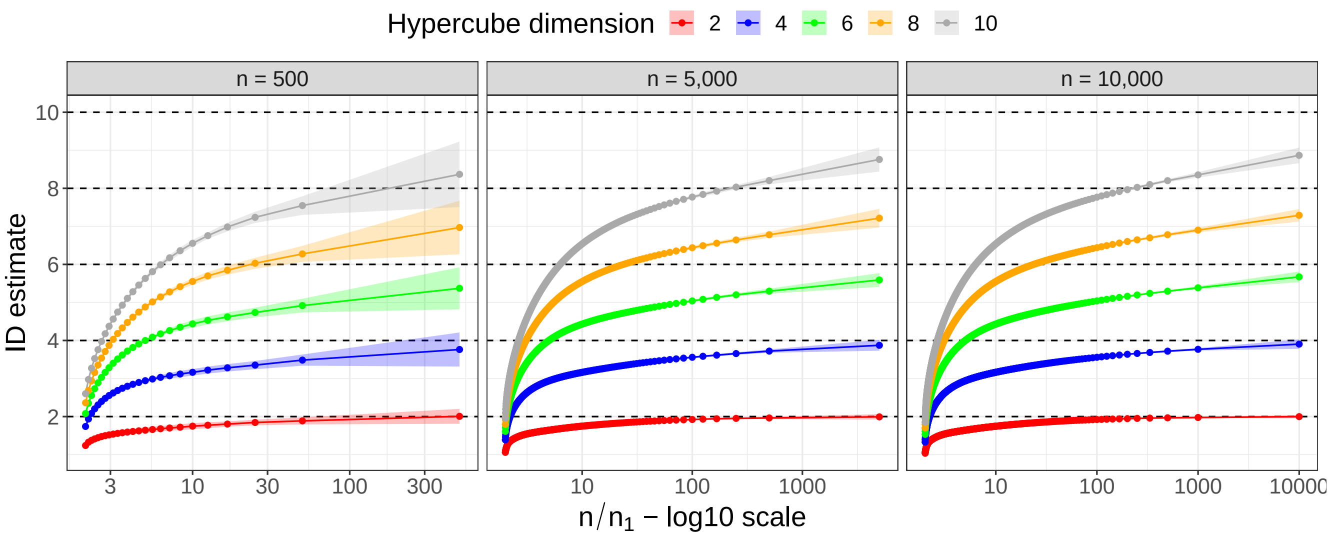

Figure S4. Evolution of the `id` estimates as a function of the ratio n/n_1 (logarithmic scale) computed on uniform hypercubes characterized by different sample sizes and increasing true id. The horizontal lines highlight the true values of the `id`.

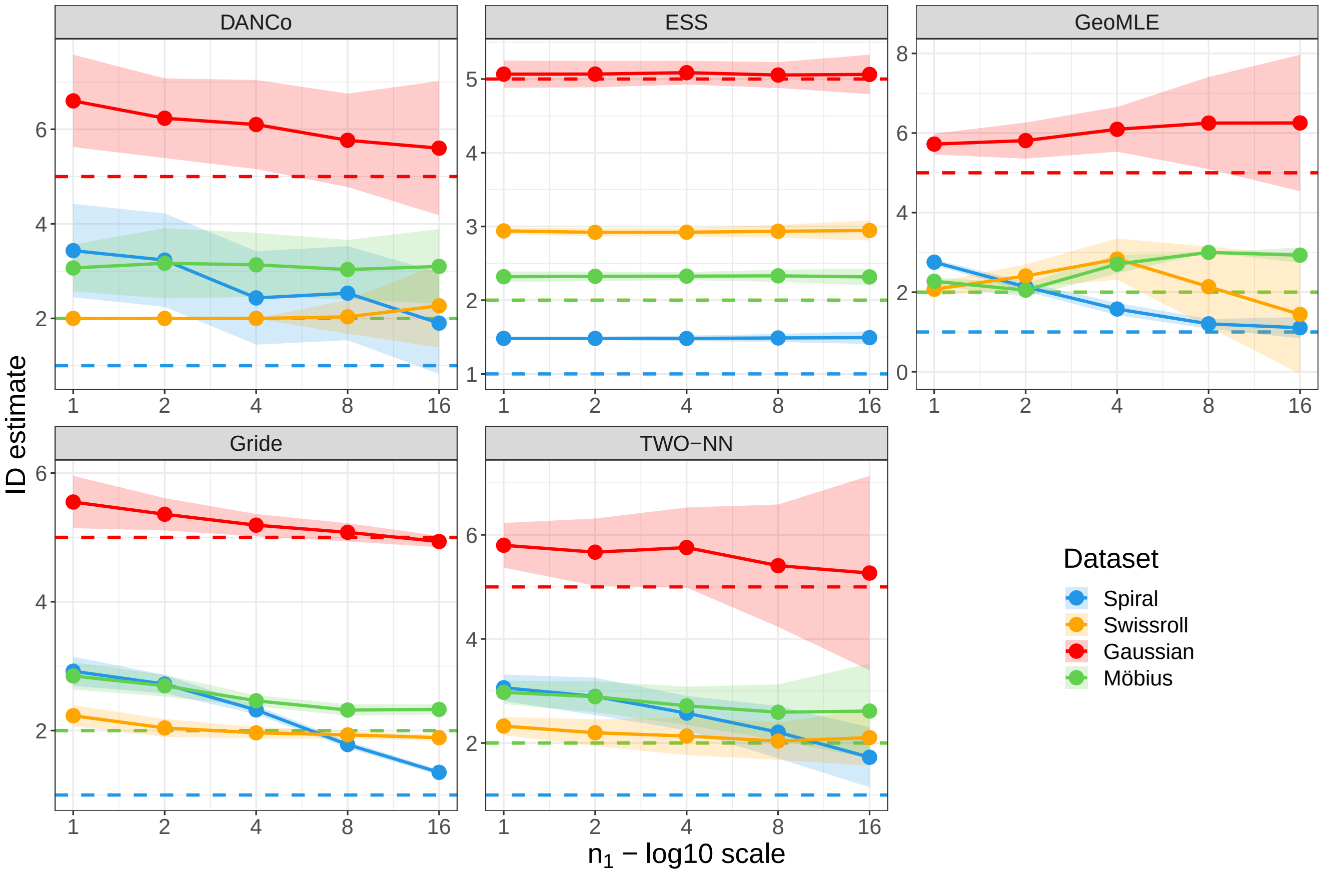

Figure S5. Evolution of the `id` estimates as a function of the lowest NN order n_1 (logarithmic scale) averaged over 30 different replicas. The horizontal lines highlight the true values of the `id`, and the shaded areas show the standard error of the estimates.

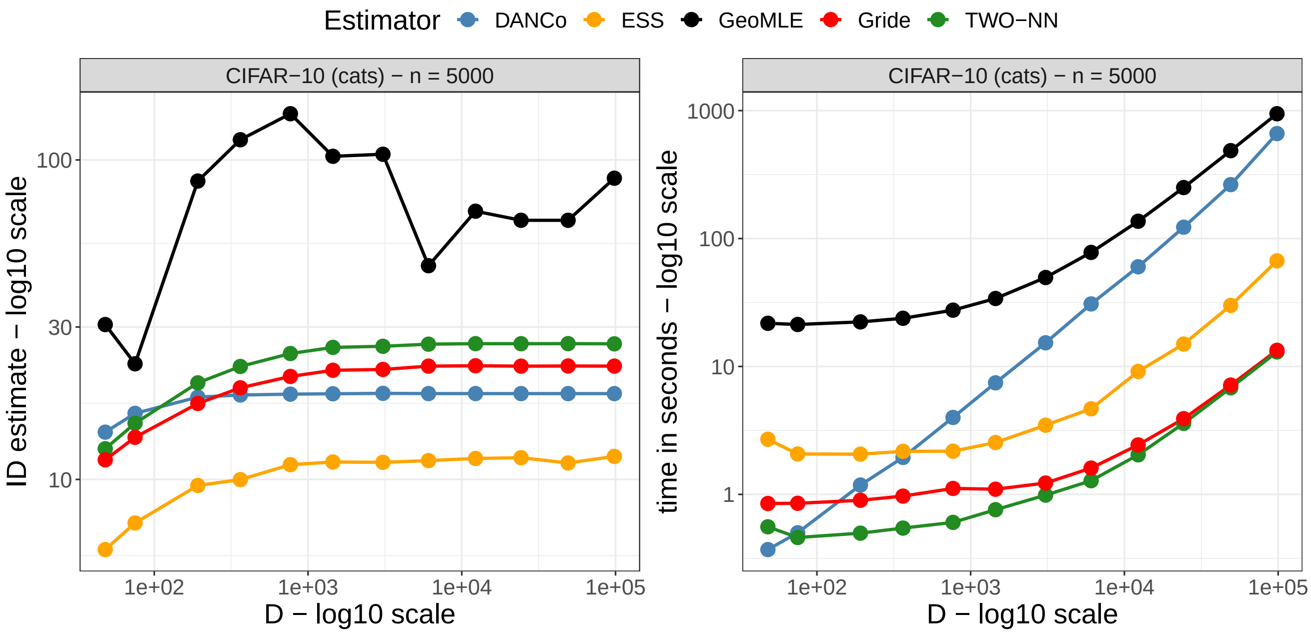

Figure S6. Trajectories of estimated `ids` (left panel) and elapsed times in seconds (right panel) obtained on the `CIFAR-10 (cat)` dataset with `DANCo`, `GeoMLE`, `ESS`, `TWO-NN`, and `Gride`. The number of feature D is defined as $D = 3q^2$, where $q \in \{8, 11, 16, 22, 32, 45, 64, 90, 128, 182\}$.

5 Additional simulation studies

5.1 `Gride` and the NN order ratios

The dataset considered here is the same used to create Figure 6 in the main paper. It contains 50000 data points from a two-dimensional Gaussian distribution and perturb them with orthogonal Gaussian white noise. Recall that we are comparing the results obtained in two cases: one-dimensional (1D) and twenty-dimensional (20D) noise; in both cases, the perturbation variance is set to $\sigma^2 = 0.0001$. Here, we consider several `Gride` models by changing the ratio $n_{2,1} = n_2/n_1$ of the order of the nearest neighbors used to compute $\dot{\mu}$. The results are shown in Figure S7. On the x-axis we report the mean neighbor distance computed as $\bar{r} = (r_{n_2} + r_{n_1})/2$, averaged over all the observations.

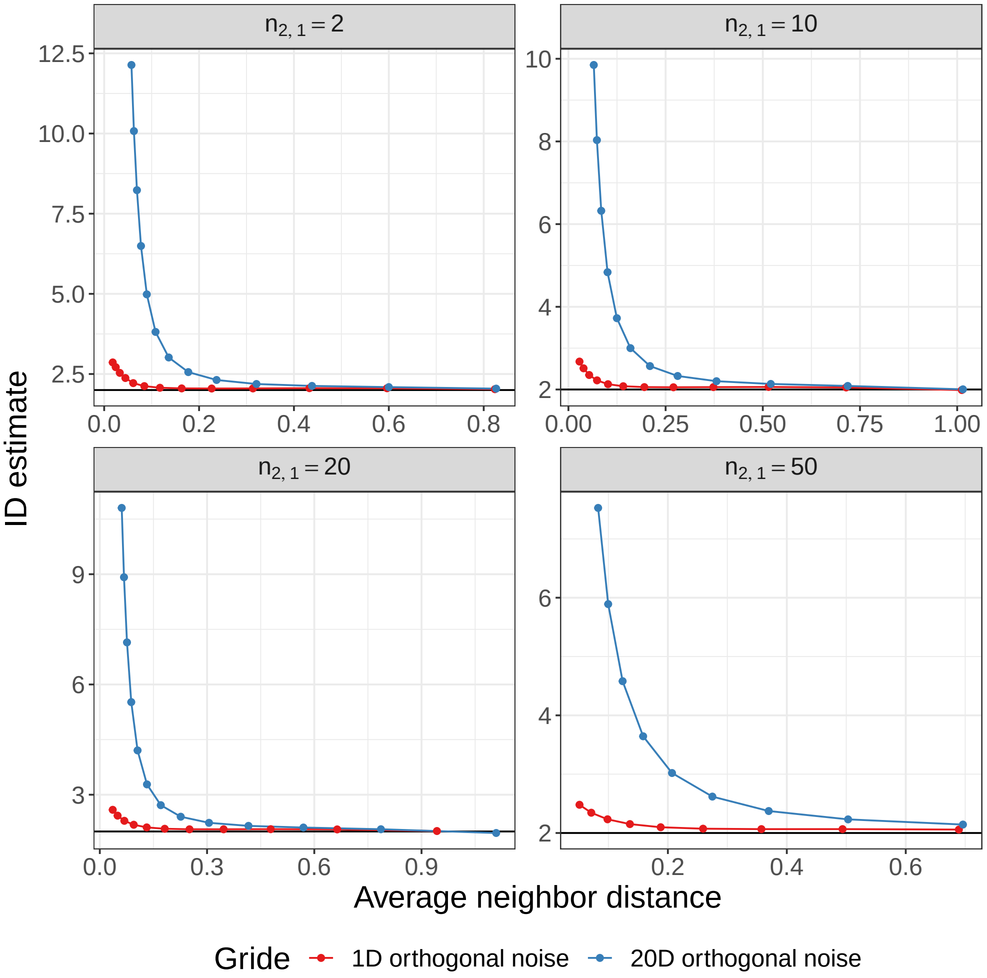

Figure S7. Analysis of the impact of the scale on the `id` estimates for different `Gride` models performed on a 2D noisy Gaussian dataset. The `id` is calculated maximizing the likelihood of Equation (10); the errorbars computed with the Fisher information are smaller than the marker size.

Note that the first points in the top left panel corresponds to the `TWO-NN` estimates. When $\bar{r}$ is of the same order as σ, the `id` estimated by the `Gride` models is much higher than 2, the true value. For instance, when $\bar{r} \approx \sigma$, the geometry of the neighborhoods is approximately 3-dimensional, and, consistently, `id` ≈ 2.85 (red line, top left panel of Figure S7). As we increase the range of distances involved in the estimate, all the models display a plateau around `id` ≈ 2. However, when $n_{2,1} = 2$, the `id` stabilizes around two at smaller scales for low and high dimensional noise. Indeed, the red lines in the different panels show that `id` ≈ 2.1 at $\bar{r} \approx 0.08$ for $n_{2,1} = 2$, at $\bar{r} \approx 0.14$ for $n_{2,1} = 20$ and $\bar{r} \approx 0.18$ for $n_{2,1} = 50$. Similarly, for the blue lines we have `id` ≈ 2.2 at $\bar{r} \approx 0.2$ for $n_{2,1} = 2$, at $\bar{r} \approx 0.45$ for $n_{2,1} = 20$ and $\bar{r} \approx 0.6$ for $n_{2,1} = 50$. A broader plateau makes it easier to identify the number of relevant directions present in the dataset. Therefore, our numerical experiments suggest that the choice $n_{2,1} = 2$ is the most appropriate in practical applications.

5.2 Robustness to noise

Here, we present the complete results of the last simulation study described in Section 3.1.3 of the main paper. From the panels in Figures S8, we observe that the estimators present similar patterns for both the `id` values we considered. In the two scenarios where no noise is added to the data (two panels in the first column), `MG` and `Gride` obtain very good results, being both close to the true `id` for almost every value of n_1. This implies that, in the absence of noise, even the `TWO-NN` estimator is a reliable method. However, when the size of the neighborhood increases considerably, `Gride` tends to underestimate the value $d = 5$ (first two panels in the bottom row). This effect is mostly caused by the limited sample size, which in turn leads to violations of the underlying hypotheses. The underestimation disappears when larger sample sizes are considered. As expected, a consistent increment in the `id` estimates appears as more noise is added to the data. For small neighborhoods, `Gride` and `MG` show similar behaviors, while as n_1 increases `MG` tends to perform similarly to `LB`. `Gride` instead decreases faster than the two competitors. The results suggest that our proposal is more robust than the two model-based competitors when handling noisy datasets.

References

1. Facco, E., D'Errico, M., Rodriguez, A. & Laio, A. Estimating the intrinsic dimension of datasets by a minimal neighborhood information. *Sci. Reports* **7**, 1–8, DOI: 10.1038/s41598-017-11873-y (2017).

2. Kingman, J. F. C. *Poisson Processes.*, vol. 3 (Oxford Science Publications, 1992).

3. Moltchanov, D. Distance distributions in random networks. *Ad Hoc Networks* **10**, 1146–1166, DOI: 10.1016/j.adhoc.2012.02.005 (2012).

4. Facco, E. & Laio, A. *The intrinsic dimension of biological data landscapes*. Ph.D. thesis, SISSA, Trieste, ITA (2017).

5. Ansuini, A., Laio, A., Macke, J. H. & Zoccolan, D. Intrinsic dimension of data representations in deep neural networks. *Adv. Neural Inf. Process. Syst.* **32** (2019). 1905.12784.

6. Allegra, M., Facco, E., Denti, F., Laio, A. & Mira, A. Data segmentation based on the local intrinsic dimension. *Sci. Reports* **10**, 1–27, DOI: 10.1038/s41598-020-72222-0 (2020). 1902.10459.

7. McFadden, D. Modeling the Choice of Residential Location. *Transp. Res. Rec.* **672**, 75–96 (1978).

8. Amsaleg, L. *et al.* Extreme-value-theoretic estimation of local intrinsic dimensionality. *Data Min. Knowl. Discov.* **32**, 1768–1805, DOI: 10.1007/s10618-018-0578-6 (2018).

9. Houle, M. E. Dimensionality, Discriminability, Density & Distance Distributions. *ICDMW* (2013).

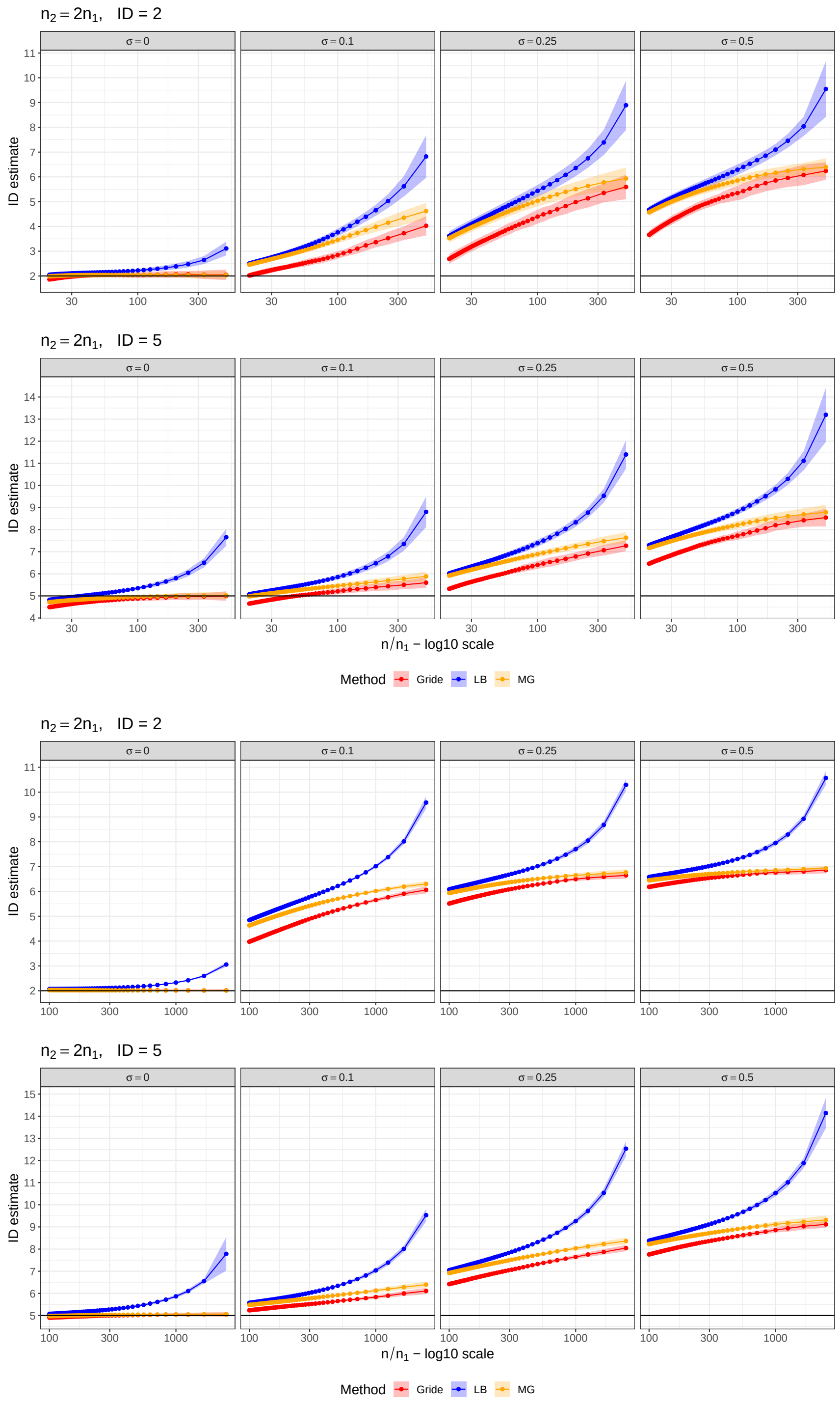

Figure S8. Each panel shows the average estimates of the `id` over 30 replicates with three different methods: `Gride`, `LB`, and `MG`. The samples size of each replica is 1000 for the top two rows, and 5000 for the bottom two rows. The confidence bands are drawn at ± 2 standard errors. In the panels showcased in rows 1 and 3, the true `id` is $d = 2$, in the panels in rows 2 and 4 the true `id` is $d = 5$.
